# Transient ATM inhibition enhances knock-in efficiency in hematopoietic stem cells by attenuating the DNA damage response

**DOI:** 10.1073/pnas.2531364123

**Published:** 2026-10-20

**Authors:** Munkh-Erdene Natsagdorj, Hiromasa Hara, Kohei Iida, Yuji Kashiwakura, Nemekhbayar Baatartsogt, Enkhzaya Ganzorig, Daniele Canarutto, Sandra Pinho, Luigi Naldini, Tsukasa Ohmori, Yutaka Hanazono, Fumio Nakahara

**Affiliations:** ^a^ Division of Regenerative Medicine, Center for Molecular Medicine, Jichi Medical University, Shimotsuke 329-0498, Tochigi, Japan; ^b^ Laboratory of Regenerative and Cellular Medicine, Center for Development of Advanced Medical Technology, Jichi Medical University, Shimotsuke 329-0498, Tochigi, Japan; ^c^ Division of Animal Resource Development, Center for Development of Advanced Medical Technology, Jichi Medical University, Shimotsuke 329-0498, Tochigi, Japan; ^d^ Center for Experimental Medicine, Jichi Medical University, Shimotsuke 329-0498, Tochigi, Japan; ^e^ Department of Biochemistry, School of Medicine, Jichi Medical University, Shimotsuke 329-0498, Tochigi, Japan; ^f^ San Raffaele Telethon Institute for Gene Therapy, Istituto di Ricovero e Cura a Carattere Scientifico San Raffaele Scientific Institute, Milan 20132, Italy; ^g^ Faculty of Medicine and Surgery, Faculty of Medicine and Surgery, Vita-Salute San Raffaele University, Milan 20132, Italy; ^h^ Department of Pharmacology and Regenerative Medicine, University of Illinois at Chicago, Chicago, IL 60612; ^i^ Translational Research Laboratory, Center for Development of Advanced Medical Technology, Jichi Medical University, Shimotsuke 329-0498, Tochigi, Japan

**Keywords:** hematopoietic stem cells, genome editing, knock-in, Ataxia-telangiectasia mutated, DNA damage response

## Abstract

Precise genome editing in hematopoietic stem cells (HSCs) holds great potential for treating inherited blood disorders; however, low knock-in (KI) efficiency and loss of functional HSCs after genome editing remain major obstacles. Here, we demonstrate that transient inhibition of Ataxia-Telangiectasia Mutated (ATM) kinase markedly enhanced KI efficiency in mouse HSCs genome-edited with Cas9/ribonucleoprotein (RNP) and AAV donor DNA. Phosphoproteomic and capillary western blot analyses revealed that ATM inhibition suppressed aberrant Cas9-AAV-induced ATM activation and the subsequent DNA damage response, reduced p53-dependent apoptosis, and enhanced survival of KI HSCs. In serial transplantation assays, ATM inhibition preserved long-term engrafting KI HSCs, increasing their frequency from ~0.3 to 40% in secondary recipients, an over 100-fold improvement. Furthermore, in an X-linked severe combined immunodeficient mouse model, ATM inhibition increased KI efficiency and restored T- and B cell differentiation. Comprehensive safety analyses, including long-read nanopore sequencing, amplicon deep sequencing, whole genome sequencing and clonal tracking, did not detect increased genomic alterations, chromosomal abnormalities, or clonal expansion following transient ATM inhibition. Taken together, these findings establish transient ATM inhibition as an effective strategy to enhance KI-mediated genome editing while maintaining the long-term repopulating capacity of HSCs.

## Significance

We demonstrate that transient Ataxia-Telangiectasia Mutated (ATM) inhibition significantly enhances precise gene integration (knock-in) efficiency in HSCs by suppressing excessive DNA damage response and apoptosis. This approach preserves the long-term repopulating capacity of genome-edited HSCs and enables effective genetic correction in disease models. These strikingly findings offer a promising strategy for safe and efficient therapeutic genome editing in HSCs.

Hematopoietic stem cells (HSCs), defined by their capacity for self-renewal and multilineage differentiation, are essential for the lifelong hematopoietic maintenance (1, 2). Allogeneic HSC transplantation (HSCT) remains the standard treatment for a broad range of hematological malignancies and inherited blood disorders, including severe combined immunodeficiency (SCID), β-thalassemia, and sickle cell disease (SCD) (3–5). Despite its curative potential, HSCT is limited by the shortage of HLA-matched donors and the risk of graft-versus-host disease (6, 7). To overcome these challenges, gene therapy approaches have been developed to enable autologous correction of genetic defects (8–10). However, most current strategies rely on viral vector-mediated random integration of therapeutic transgenes, which can result in variable expression and the potential for insertional mutagenesis (11).

In contrast, ex vivo genome editing, followed by autologous transplantation, offers a more precise alternative, enabling targeted gene modification of disease-causing mutations (12). The recent clinical success of Casgevy^®^—a CRISPR/Cas9-based therapy that disrupts the erythroid-specific enhancer of *BCL11A* via nonhomologous end joining (NHEJ) to induce fetal hemoglobin expression—demonstrated proof of concept and therapeutic benefit in patients with β-thalassemia and SCD (13, 14), even though the underlying causative mutation remains uncorrected. While Casgevy^®^ represents a major advance, its gene disruption strategy is limited to disorders with well-defined repressor elements and compensatory pathways. For the broader range of monogenic hematologic diseases, precise gene correction at endogenous loci is required, necessitating efficient knock-in (KI)-based genome editing (15, 16). KI is typically achieved through homology-directed repair (HDR), a pathway essential for precise gene correction (17). However, KI efficiency in HSCs remains limited due to their quiescent state and preference for error-prone NHEJ, which restricts the application of genome editing as a generalizable therapeutic platform. Multiple strategies have been explored to enhance KI efficiency in HSCs, including ex vivo culture systems to stimulate HSC proliferation (18), generation of spacer-nick to prevent NHEJ (19), pharmacological inhibition of NHEJ factors such as DNA Ligase IV (20, 21), DNA-dependent protein kinase catalytic subunit (DNA-PKcs) (22) or inhibition of p53-binding protein 1 (53BP1) (23), which competes with HDR and promotes error-prone repair. Although these approaches improve editing efficiency, concerns remain regarding their potential effects on HSC function, including disruption of stem cell homeostasis and long-term engraftment (24). Therefore, alternative strategies to promote KI without impairing HSC properties are needed to advance genome editing for therapeutic application.

Ataxia-telangiectasia mutated (ATM) kinase functions as an early sensor of DNA double-strand break (DSB) and is a central regulator of the DNA damage response (DDR), coordinating the balance between DNA repair and apoptosis. Upon activation, ATM phosphorylates substrates, including p53, H2AX, and 53BP1, which collectively influence repair pathway choice and cell fate (25–27). Previously, we found that linear donor DNA, regardless of strand configuration, triggered excessive ATM activation, whereas plasmid donors did not in mouse cell lines (28). Importantly, transient inhibition of ATM significantly enhanced KI efficiency in these cell lines when Cas9 ribonucleoprotein (RNP) was used with AAV donor templates. Based on these findings, we extend this strategy to primary HSCs. Given the unique DNA repair characteristics of HSCs and their sensitivity to genotoxic stress, we hypothesized that transient ATM inhibition could similarly modulate the DDR to favor KI while preserving stemness. To test this hypothesis, we combined Cas9/RNP and AAV donor DNA in ex vivo cultured mouse HSCs, with or without transient ATM inhibition. We then assessed the impact on KI efficiency, DDR signaling, and functional stem cell activity using transplantation-based assays. These data revealed that transient ATM inhibition improves the precision and therapeutic potential of HSC genome editing.

## Results

### Transient ATM Inhibition Enhances KI Efficiency in Hematopoietic Cell Lines.

**Fig. 1.**
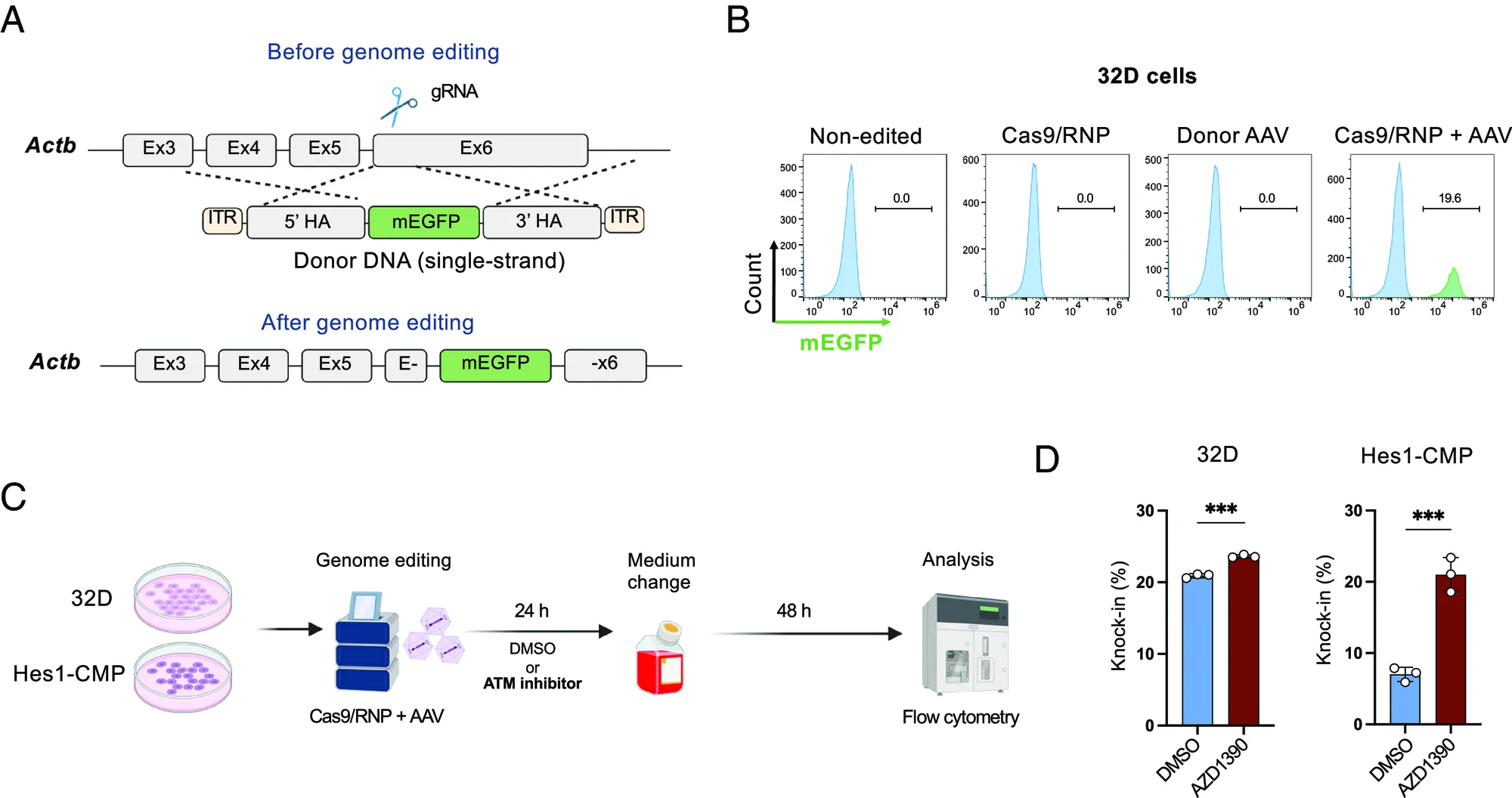
Transient ATM inhibition enhances knock-in (KI) efficiency in cell lines. (*A*) Diagram of the mEGFP KI construct. (*B*) Representative flow cytometry results. When mEGFP is knocked-in into *Actb* in 32D cells, the mEGFP expression is detected by flow cytometry. (*C*) Schematic of the experimental procedure for genome editing in cell lines. (*D*) KI efficiency following 10 nM AZD1390 treatment in 32D cells and Hes1-CMP cells. Data are presented as the mean ± SD (n = 3). Unpaired *t* tests were conducted. For 32D cells, *P* = 0.0002; for Hes1-CMP cells *P* = 0.0005.

First, we tested whether transient ATM inhibition enhances KI efficiency in hematopoietic cells, using a fluorescent-based KI reporter system. A promoterless monomeric EGFP (mEGFP) coding sequence was inserted into exon 6 of the mouse *beta Actin* (*Actb)* gene using a donor DNA flanked by 1-kilobase (kb) 5′ and 3′ homology arms (HA) (Fig. 1*A*). Because the donor DNA construct lacks an exogenous promoter, mEGFP expression occurred only when the allele was precisely genome-edited via HDR, allowing accurate quantification of KI efficiency. (Fig. 1*B*).

To evaluate the effect of transient ATM inhibition on KI efficiency, we tested two mouse hematopoietic cell lines: 32D Clone 3 cell line, and hairy and enhancer of split 1 (Hes1)-transduced mouse common myeloid progenitor (Hes1-CMP) cells (29). Cells were electroporated with Cas9/RNP, transduced with AAV donor DNA, and treated for 24 h with or without 10 nM AZD1390, a potent and selective ATM inhibitor (Fig. 1*C*). Flow cytometric analysis revealed that AZD1390 treatment significantly increased the percentage of mEGFP^+^ cells in both cell lines compared to DMSO controls (Fig. 1*D*). These results indicate that transient ATM inhibition enhanced KI efficiency in hematopoietic cell lines.

### Transient ATM Inhibition Increases KI Efficiency in Ex Vivo Cultured Primary Mouse HSCs.

**Fig. 2.**
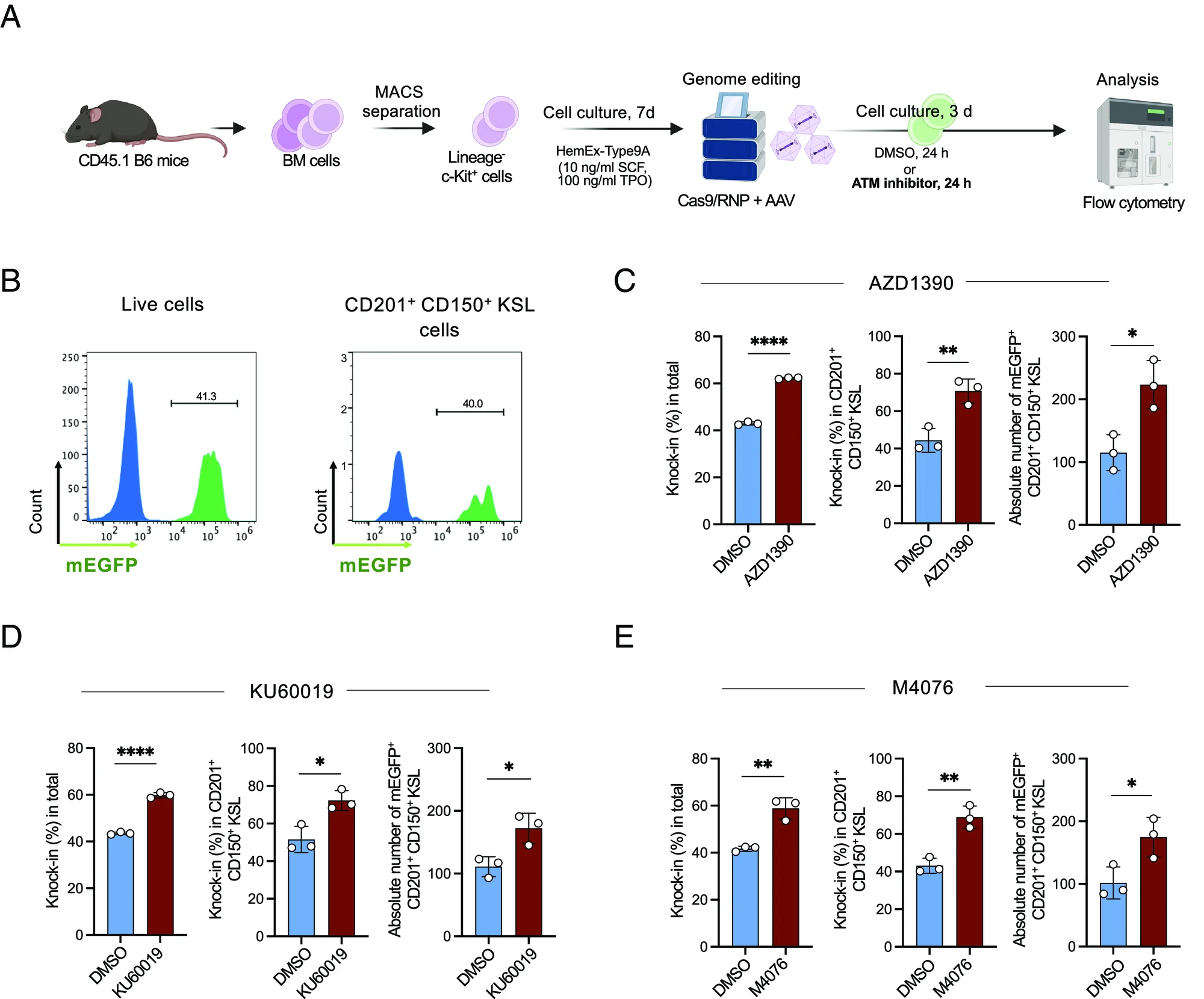
Transient ATM inhibition enhances knock-in (KI) efficiency in mouse HSCs ex vivo. (*A*) Schematic of the ex vivo genome editing protocol in mouse HSCs. (*B*) Representative flow cytometry plots. KI efficiency was assessed in live cells and CD201^+^ CD150^+^ c-Kit^+^ Sca1^+^ Lin^−^ cells (HSCs). (*C*) Genome editing results following treatment with 10 nM AZD1390. Data are presented as the mean ± SD (n = 3). KI (%) in total, unpaired *t* tests were conducted. *P* < 0.0001; For KI (%) in HSCs, *P* = 0.0082; For Absolute number of mEGFP^+^ HSCs, unpaired *t* tests were conducted. *P* = 0.0173. (*D*) Genome editing outcomes following treatment with 1 µM KU60019. Data are presented as the mean ± SD (n = 3). For KI (%) in total, unpaired *t* tests were conducted. *P* < 0.0001; For KI (%) in HSCs, unpaired *t* tests were conducted. *P* = 0.0154; For Absolute number of mEGFP^+^ HSCs, unpaired *t* tests were conducted. *P* = 0.0214. (*E*) Genome editing outcomes following treatment with 1 µM M4076. Data are presented as the mean ± SD (n = 3). For KI (%) in total, unpaired *t* tests were conducted. *P* = 0.0032; For KI (%) in HSCs, unpaired *t* test were conducted. *P* = 0.0039; For Absolute number of mEGFP^+^ HSCs, *P* = 0.0361.

We next evaluated the effect of transient ATM inhibition on genome editing in primary mouse HSCs. Lineage^−^ cKit^+^ (LK) cells were isolated from mouse bone marrow (BM) and cultured for 7 d ex vivo using HemEx-Type9A medium to promote HSCs expansion (30, 31) (Fig. 2*A*). The KI reporter described above was used to detect and quantify mEGFP-expressing cells. KI efficiency was assessed in total cultured LK cells or in phenotypically defined HSCs (CD201^+^ CD150^+^ c-Kit^+^ Sca1^+^ Lineage^-^; referred to as CD201^+^ CD150^+^ KSL) using flow cytometry (Fig. 2 *A* and *B* and *SI Appendix*, Fig. S1).

To test the effect of transient ATM inhibition, we evaluated several ATM inhibitors, including AZD1390, M4076, KU60019, AZ32, and KU55933. Overall, all compounds tested increased KI efficiency in both total cells and the HSCs (Fig. 2 *C*-*E*), with AZ32 and KU55933 being the least efficient (*SI Appendix*, Fig. S2). Among them, 10 nM AZD1390 produced the most significant effect, increasing KI efficiency from 43.0 ± 0.7 to 62.2 ± 0.4 in total cells and from 44.4 ± 6.4 to 70.7 ± 6.6% in the HSCs (*P* < 0.0001 and *P* < 0.0082, respectively). Notably, quantification of mEGFP^+^ HSCs confirmed that ATM inhibitor treatment significantly enhanced KI efficiency in the HSCs. (Fig. 2 *C*-*E*). This effect was observed in a dose-dependent manner following AZD1390 treatment (*SI Appendix*, Fig. S3). These results indicate that transient ATM inhibition significantly enhances KI efficiency in ex vivo cultured primary mouse HSCs.

### HSCs Genome-Edited With ATM Inhibitors Retain Long-Term, Multilineage Repopulating Capacity.

**Fig. 3.**
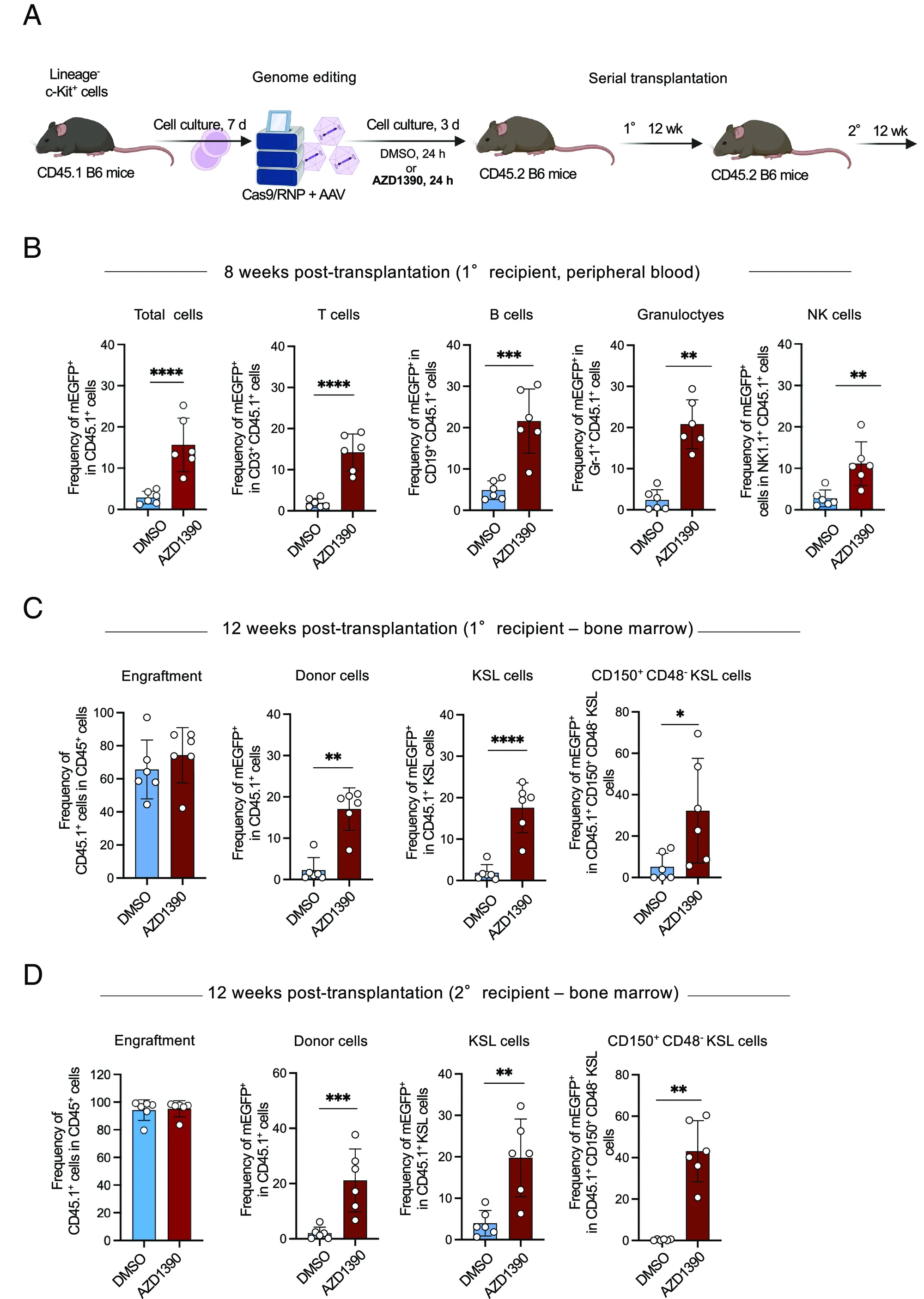
Transient ATM inhibition enhances knock-in (KI) efficiency in functional HSCs. (*A*) Schematic of the experimental procedure for the transplantation analysis. (*B*) Peripheral blood (PB) analysis at 8 wk posttransplantation (primary). Data are shown as the mean ± SD (n = 6). For frequency of mEGFP^+^ cells in donor cells, unpaired *t* test was conducted. *P* < 0.0001; For frequency of mEGFP^+^ cells in donor T cells, unpaired *t* test was conducted. *P* < 0.0001; For frequency of mEGFP^+^ cells in donor B cells, unpaired *t* test was conducted. *P* = 0.0002; For frequency of mEGFP^+^ cells in donor granulocytes, Mann–Whitney test was conducted. *P* = 0.0022; For frequency of mEGFP^+^ cells in donor NK cells, unpaired *t* test was conducted. *P* = 0.0017. (*C*) BM analysis at 12 wk posttransplantation (primary). Data are shown as the mean ± SD (n = 6). For engraftment rate, unpaired *t* test was conducted. *P* = 0.502; For frequency of mEGFP^+^ cells in donor cells, Mann–Whitney test was conducted. *P* = 0.0043; For frequency of mEGFP^+^ cells in donor KSL cells, unpaired *t* test was conducted. *P* < 0.0001; For frequency of mEGFP^+^ cells in donor CD150^+^ CD48^−^ KSL cells (HSCs), unpaired *t* test was conducted. *P* = 0.014. (*D*) BM analysis at 12 wk posttransplantation (secondary). Data are shown as the mean ± SD (n = 6). For engraftment rate, Mann–Whitney test was conducted. *P* = 0.8182; For frequency of mEGFP^+^ cells in donor cells, unpaired *t* test was conducted. *P* = 0.0005; For frequency of mEGFP^+^ cells in donor c-Kit^+^ Sca1^+^ Lin^−^ cells, unpaired *t* test was conducted. *P* = 0.0016; For frequency of mEGFP^+^ cells in donor HSCs, Mann–Whitney test was conducted. *P* = 0.0022.

To determine whether transient ATM inhibition preserves the long-term repopulating capacity of genome-edited HSCs, we performed serial transplantation using CD45.2^+^ recipient mice and CD45.1^+^ donor mice on a C57BL/6 background (Fig. 3*A*). LK cells harvested from donor BM were cultured ex vivo for 7 d, genome-edited with Cas9/RNP and AAV donor DNA with or without 10 nM AZD1390 and transplanted into lethally irradiated recipient mice (n = 6 per group).

Multilineage hematopoietic reconstitution was first assessed by complete blood count (CBC) analysis 4 wk posttransplantation. No significant differences were observed between DMSO and AZD1390-treated groups in red blood cell count (RBC), white blood cell (WBC) count, platelet count, hematocrit, or hemoglobin levels (*SI Appendix*, Fig. S4*A*). In addition, major leukocyte fractions, including T cells, B cells, NK cells, and granulocytes were analyzed in PB at 4 and 8 wk posttransplantation using flow cytometry. No significant differences were observed between the two groups (*SI Appendix*, Fig. S4 *B* and *C*). CD45.2^+^ whole BM cells were used as accessory cells in both transplantations. To specifically assess KI efficiency in donor-derived cells, CD45.1^+^ compartment was analyzed. Notably, frequency of mEGFP^+^ cells within CD45.1^+^ cells was significantly higher in AZD1390-treated mice across all major lineages, including T cells, B cells, NK cells, and granulocytes, compared with DMSO controls (Fig. 3*B* and *SI Appendix*, Fig. S5 *A* and *B*).

Importantly, AZD1390 treatment did not impact the overall BM engraftment of donor CD45.1 cells, as seen in primary and secondary recipients at 12 wk posttransplant (Fig. 3 *C* and *D*). Yet, BM analysis revealed a significantly increased frequency of mEGFP^+^ cells in AZD1390-treated mice among total CD45.1^+^ cells, CD45.1^+^ KSL cells, and CD45.1^+^ CD150^+^ CD48^−^ KSL cells (HSCs). In CD45.1^+^ HSCs specifically, the frequency of mEGFP^+^ cells was 5.1 ± 6.6% in the DMSO group versus 32.4 ± 25.3% in the AZD1390 group (*P* = 0.014) (Fig. 3*C* and *SI Appendix*, Fig. S6). This elevated frequency of mEGFP^+^ cells in the AZD1390 group was maintained in secondary recipients at 12 wk posttransplantation, reaching 43.1 ± 14.7%, whereas the DMSO group decreased to 0.33 ± 0.37% (Fig. 3*D*). These results indicate that AZD1390 treatment enhanced KI efficiency in functional HSCs without impairing multilineage hematopoietic reconstitution.

### ATM Inhibition Suppresses the Overactivation of ATM Induced by Cas9/RNP and AAV Donor DNA, Thereby Attenuating Downstream p53–Caspase-3 Activation.

**Fig. 4.**
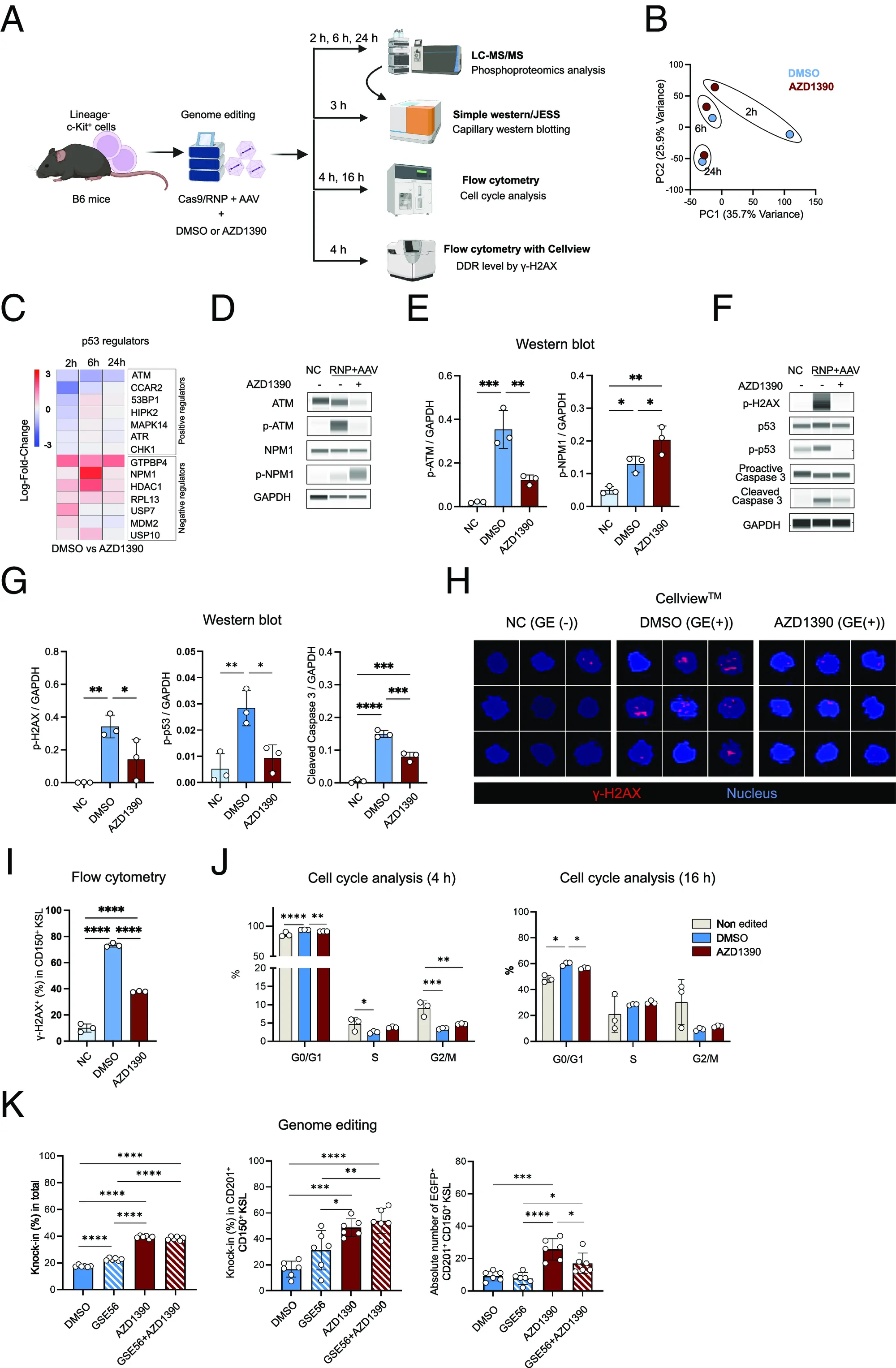
Transient ATM inhibition suppresses Cas9-AAV-induced DNA damage response (DDR) and subsequent p53-dependent apoptosis. (*A*) Schematic of the experimental procedure for phosphoproteomic analysis. (*B*) PCA results of the phosphopeptides after genome editing with Cas9/RNP and AAV donor DNA in the presence or absence of ATM inhibitor at 2-, 6-, and 24-hours postnucleofection. (*C*) Heatmap showing changes of phosphorylation level of p53 regulators at 2-, 6-, and 24-h postgenome editing, comparing DMSO and AZD1390-treated groups. Log-transformed fold changes were used. (*D*) Western blotting results showing genome editing with Cas9/RNP and AAV donor DNA induces ATM activation (Ser-1987) at 4 h postnucleofection. 10 nM AZD1390 treatment suppresses the activation of ATM and also enhances the activation of NPM1 (Ser-10). (*E*) Quantification of the western blot results. The signal intensities were normalized to GAPDH. Data are presented as the mean ± SD (n = 3). For p-ATM, Tukey’s multiple comparisons tests were conducted. NC vs. DMSO, *P* = 0.0005; NC vs. AZD1390, *P* = 0.1144; DMSO vs. AZD1390, *P* = 0.0038. For p-NPM1, Tukey’s multiple comparisons tests were conducted. NC vs. DMSO, *P* = 0.035; NC vs. AZD1390, *P* = 0.0016; DMSO vs. AZD1390, *P* = 0.0477. (*F*) Western blotting results showing Cas9/RNP and AAV donor DNA genome editing activates H2AX (Ser-139), p53 (Ser-15), and Caspase 3. 10 nM AZD1390 treatment suppresses the activation of H2AX, p53, and following Caspase 3. (*G*) Quantification of the western blot results. The signal intensities were normalized to GAPDH. Data are presented as the mean ± SD (n = 3). For p-H2AX, Šídák’s multiple comparisons tests were conducted. NC vs. DMSO, *P* = 0.0065; NC vs. AZD1390, *P* = 0.0799; DMSO vs. AZD1390, *P* = 0.0468. For p-p53, Tukey’s multiple comparisons tests were conducted. NC vs. DMSO, *P* = 0.0073; NC vs. AZD1390, *P* = 0.6937; DMSO vs. AZD1390, *P* = 0.0179. For Cleaved Caspase 3, Tukey’s multiple comparisons tests were conducted. NC vs. DMSO, *P* < 0.0001; NC vs. AZD1390, *P* = 0.0003; DMSO vs. AZD1390, *P* = 0.0005. (*H*) Representative images of flow cytometry analysis with Cellview™. The AZD1390 treatment decreases the γ-H2AX^+^ cells compared to DMSO control. GE stands for genome editing with Cas9/RNP and AAV donor DNA. (*I*) Flow cytometry quantification of the γ-H2AX activation analysis. Data are presented as the mean ± SD (n = 3). Tukey’s multiple comparisons tests were conducted. NC vs. DMSO, *P* < 0.0001; NC vs. AZD1390, *P* < 0.0001; DMSO vs. AZD1390, *P* < 0.0001. (*J*) Quantification of cell cycle distribution. Cell cycle analysis at 4 h postnucleofection: Data are presented as the mean ± SD (n = 3). For G0/G0, Tukey’s multiple comparisons tests were conducted. NC vs. DMSO, *P* < 0.0001, NC vs. AZD1390, *P* = 0.0088, DMSO vs. AZD1390, *P* = 0.0830. For S, Tukey’s multiple comparisons tests were conducted. NC vs. DMSO, *P* = 0.0449, NC vs. AZD1390, *P* = 0.69, DMSO vs. AZD1390, *P* = 0.2054. For G2/M, Tukey’s multiple comparisons tests were conducted. NC vs. DMSO, *P* = 0.0002, NC vs. AZD1390, *P* = 0.0038, DMSO vs. AZD1390, *P* = 0.3519. Cell cycle analysis at 16 h postnucleofection: Data are presented as the mean ± SD (n = 3). For G0/G0, Tukey’s multiple comparisons tests were conducted. NC vs. DMSO, *P* = 0.0336, NC vs. AZD1390, *P* = 0.0675, DMSO vs. AZD1390, *P* = 0.0351. (*K*) Genome editing outcomes following treatment with 150 µg/mL GSE56 or 10 nM AZD1390 or combined. Data are presented as the mean ± SD (n = 6). For KI (%) in total, Tukey’s multiple comparisons tests were conducted. DMSO vs. GSE56, *P* < 0.0001; DMSO vs. AZD1390, *P* < 0.0001; DMSO vs. GSE56+AZD1390, *P* < 0.0001; GSE56 vs. AZD1390, *P* < 0.0001; GSE56 vs. GSE56+AZD1390, *P* = 0.3583; AZD1390 vs. GSE56+AZD1390, *P* < 0.0001. For KI (%) in HSCs, Tukey’s multiple comparisons tests were conducted. DMSO vs. GSE56, *P* = 0.0972; DMSO vs. AZD1390, *P* = 0.0002; DMSO vs. GSE56+AZD1390, *P* < 0.0001; GSE56 vs. AZD1390, *P* = 0.0462; GSE56 vs. GSE56+AZD1390, *P* = 0.0083; AZD1390 vs. GSE56+AZD1390, *P* = 0.8575. For absolute number of mEGFP^+^ CD150^+^ CD201^+^ KSL cells, Tukey’s multiple comparisons tests were conducted. DMSO vs. GSE56, *P* = 0.9572; DMSO vs. AZD1390, *P* = 0.0001; DMSO vs. GSE56+AZD1390, *P* = 0.07; GSE56 vs. AZD1390, *P* < 0.0001; GSE56 vs. GSE56+AZD1390, *P* = 0.0247; AZD1390 vs. GSE56+AZD1390, *P* = 0.0396.

To investigate the mechanism by which transient ATM inhibition increases KI efficiency in HSCs, phosphoproteomic analysis was performed. 7-d cultured LK cells were genome-edited and treated with either DMSO or 10 nM AZD1390. Samples were prepared for phosphoproteomics analysis at 2, 6, and 24 h after genome editing (Fig. 4*A*). Principal component analysis (PCA) of phosphopeptide signatures showed a clear separation between the DMSO and AZD1390 groups at the 2-h time-point, indicating distinct ATM signaling profiles after inhibition (Fig. 4*B*).

Phosphopeptide analysis revealed that p53 regulators shifted from an activated state in the DMSO group to a suppressed state in the AZD1390 group at 2 and 6 h postnucleofection. Interestingly, Nucleophosmin 1 (NPM1), a multifunctional nucleolar protein involved in both p53 regulation and genomic stability in hematopoietic cells, showed increased phosphorylation at Ser10 in the AZD1390 group. (Fig. 4*C*). We validated these findings by performing capillary western blotting at 3 to 4 h postnucleofection, between the 2- and 6-h time points. In the DMSO group, ATM was strongly activated at Ser1987, which was significantly suppressed by AZD1390 treatment. In addition, phosphorylated NPM1 at Ser10 was significantly increased in the AZD1390 group, a modification important for genomic stability (Fig. 4 *D* and *E*).

We next examined p53 and subsequent activation of Caspase-3. In the DMSO group, we observed activation of p53 and subsequent activation of Caspase-3, both of which were suppressed by AZD1390 treatment (Fig. 4 *F* and *G*). Moreover, activation of H2AX, a key downstream marker of DDR, was detected in the DMSO group, and this activation was suppressed by the AZD1390 treatment. These results were validated by flow cytometry analysis with Cellview (Fig. 4 *H* and *I* and *SI Appendix*, Fig. S7).

We next investigated whether transient ATM inhibition affected the ATM–CHK2–p21 cell-cycle checkpoint pathway. Western blot analysis demonstrated that genome editing with Cas9/RNP and AAV donor DNA induced phosphorylation of CHK2 (Thr68) and subsequent upregulation of p21 at 4 h after genome editing, both of which were effectively suppressed by 10 nM AZD1390 treatment (*SI Appendix*, Fig. S8). Because the ATM–CHK2–p21 signaling axis plays a central role in DNA damage-induced cell-cycle checkpoint regulation, we analyzed cell-cycle distribution at 4 and 16 h after nucleofection (Fig. 4*J*). No substantial differences in cell-cycle distribution were observed between DMSO- and AZD1390- groups at either time point, except for a modest reduction in the G0/G1 population. Importantly, AZD1390 treatment did not increase the proportion of cells in the HDR-permissive S or G2/M phases.

To further investigate the role of p53 in genome editing, we evaluated the dominant-negative p53 inhibitor GSE56, which has previously been reported to enhance KI efficiency in human HSCs (32). GSE56 was added to the nucleofection mixture at a final concentration of 150 μg/mL, and its effects were examined in our mouse HSC genome-editing system. DMSO-, AZD1390-, and GSE56-treated cells were included as comparison groups, and an additional group receiving both GSE56 and AZD1390 was used to evaluate the functional relationship between transient ATM inhibition and p53 suppression. Consistent with the previous findings in human HSCs, GSE56 significantly increased KI efficiency in mouse LK cells compared with the DMSO group, confirming its beneficial effect in our mouse HSC model. However, AZD1390 produced a significantly greater increase in both the frequency and absolute number of KI HSCs than GSE56 (Fig. 4*K*). Furthermore, combined treatment with GSE56 and AZD1390 did not further enhance either parameter compared with AZD1390 treatment alone. These findings suggest that attenuation of p53 signaling contributes to improved KI efficiency, whereas transient ATM inhibition may provide additional benefits beyond p53 suppression alone. Additional analyses of total cell expansion and overall genome-editing outcomes are presented in SI *Appendix F*ig. S9. These supplementary analyses showed trends consistent with those observed in HSCs and further support the conclusion that attenuation of p53 signaling contributes substantially to enhanced KI efficiency, while transient ATM inhibition provides additional benefits beyond p53 suppression alone. Consistent with this possibility, phosphorylation of 53BP1, a downstream effector of ATM and regulator of DNA DSB repair, was modestly increased following genome editing in the DMSO group and was restored toward baseline by AZD1390 treatment (*SI Appendix*, Fig. S10).

Collectively, these findings indicate that the beneficial effects of transient ATM inhibition are unlikely to be attributable to altered cell-cycle distribution. Rather, transient ATM inhibition suppresses excessive Cas9-AAV-mediated DNA damage signaling and attenuates p53-dependent apoptosis, while also exerting additional effects beyond p53 attenuation that contribute to enhanced KI efficiency in HSCs.

### Transient ATM Inhibition Enhances the Efficiency of Therapeutic Genome Editing to Correct the *Il2rg* Mutation in the X-Linked Severe Combined Immunodeficiency (X-SCID) Mouse Model.

We next evaluated whether our optimized genome editing approach, in combination with transient ATM inhibition, could effectively correct the *Il2rg* mutation, which encodes Interleukin 2 receptor subunit gamma, responsible for X-SCID. We used a previously established X-SCID mouse model carrying a single-nucleotide insertion in exon 4 of *Il2rg*, which causes a frameshift mutation and loss of IL2RG (CD132) function (33).

**Fig. 5.**
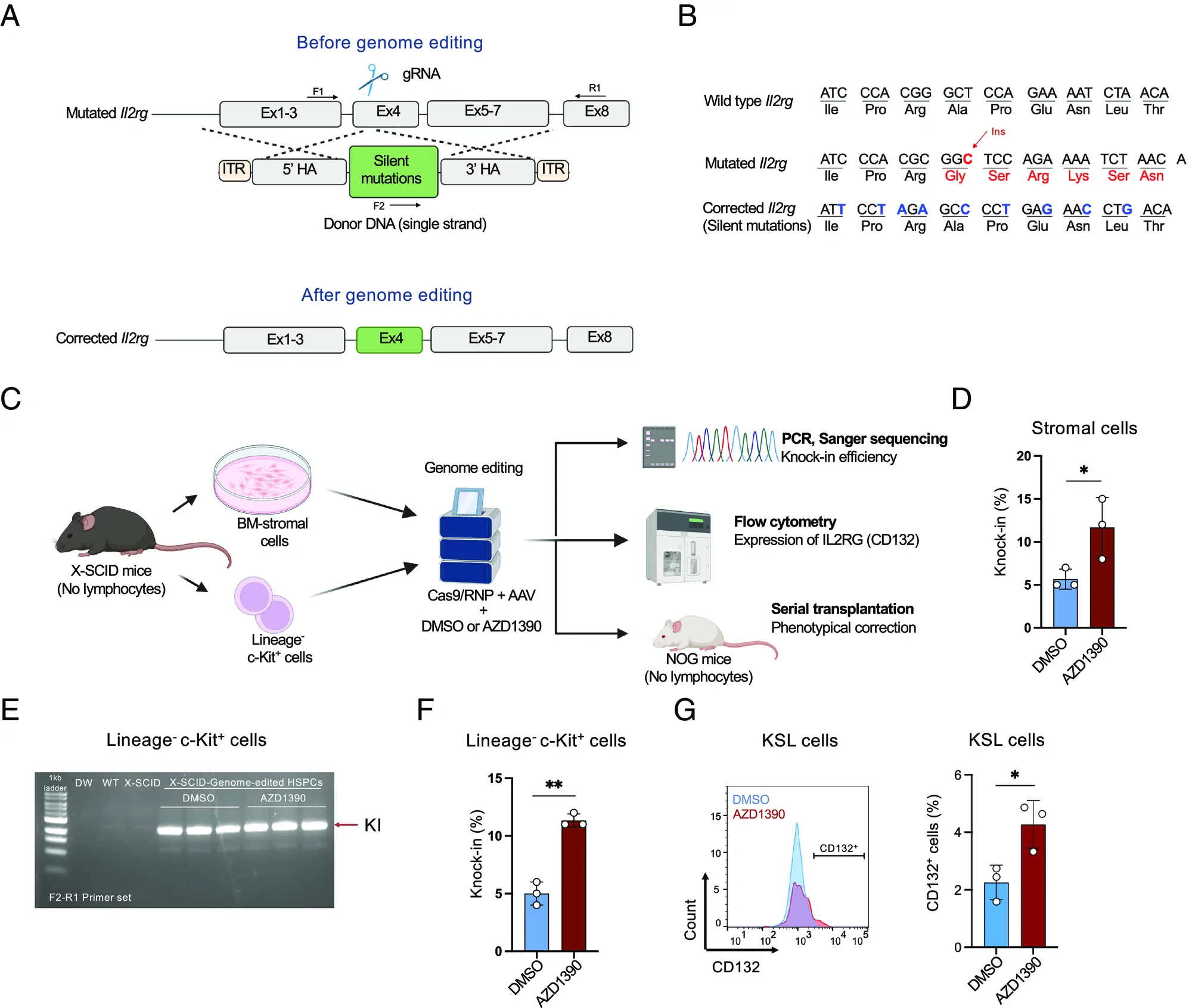
Transient ATM inhibition enhances knock-in (KI) efficiency in X-SCID mouse-derived HSPCs. (*A*) Diagram of the genome editing construct for X-SCID treatment. (*B*) Illustration of “silent mutation” strategy used to repair the *Il2rg* mutation. (*C*) Schematic of the experimental procedure for gene correction targeting the *Il2rg* mutation. (*D*) KI efficiency in BM-derived stromal cells treated with 10 nM AZD1390 treatment. Data are presented as the mean ± SD (n = 3). Unpaired *t* tests were conducted. *P* = 0.039. (*E*) Gel electrophoresis results demonstrating successful KI in X-SCID mouse BM-derived HSPCs. (*F*) KI efficiency following 10 nM AZD1390 treatment in Lineage^−^ c-Kit^+^ cells. Data are presented as the mean ± SD (n = 3). Unpaired *t* tests were conducted. *P* = 0.0012. (*G*) Representative flow cytometry histograms showing CD132 (IL2RG) expression in DMSO and AZD1390-treated groups. Frequency of CD132^+^ cells following treatment of 10 nM AZD1390. Data are presented as the mean ± SD (n = 3). Unpaired *t* tests were conducted. *P* = 0.0009.

To repair the mutation, we designed a gene correction strategy using AAV donor DNA containing silent mutations at the DSB site, flanked by 2 kb HAs at both the 5′ and 3′ ends (Fig. 5*A*). Precise KI restored the native amino acid sequence and functional protein expression (Fig. 5*B*). Before applying this approach to hematopoietic cells, we validated the genome editing system in X-SCID mouse BM–derived stromal cells immortalized with SV40 large T antigen. These cells were genome-edited with Cas9/RNP and AAV donor DNA and cultured for 5 d. PCR amplification using a primer pair spanning the 5′ HA (F1) and a downstream region outside the 3′ HA (R1) was followed by Sanger sequencing. KI efficiency was quantified using ICE CRISPR Analysis (Fig. 5*C* and *SI Appendix*, Fig. S11). Treatment with 10 nM AZD1390 significantly increased KI frequency compared to DMSO control (Fig. 5*D*).

We then applied the same protocol to 7-d ex vivo–cultured LK cells from X-SCID mice. To confirm KI at the *Il2rg* mutation site, primer pair (F2–R1) was designed to incorporate only the region of silent mutations and downstream genomic sequence. Gel electrophoresis of PCR amplicons showed successful KI in LK cells (Fig. 5*E*). Sanger sequencing and ICE analysis revealed significantly enhanced KI efficiency in the AZD1390 group compared with the controls (Fig. 5*F*). Finally, we assessed CD132 expression in KSL cells using flow cytometry. Although CD132 expression is typically low in KSLs, AZD1390-treated cells exhibited slightly higher CD132 expression levels than DMSO-treated cells and an increased frequency of CD132^+^ cells (Fig. 5*G*), indicating successful functional correction of the *Il2rg* mutation.

To evaluate the long-term repopulating capacity and functional correction of genome-edited X-SCID HSCs, AZD1390-treated LK cells were transplanted into sublethally irradiated NOG recipient mice (2.5 Gy), followed by serial transplantation (Fig. 5*C*). BM analysis showed a higher engraftment rate in AZD1390 group (52.84 ± 14.89%) than DMSO group (25.65 ± 11.67%) (*SI Appendix*, Fig. S12*A*). Amplicon deep sequencing revealed that KI efficiencies in donor-derived reads were 1.14 ± 2.2% in the DMSO group and 2.03 ± 3.78% in the AZD1390 group (*SI Appendix*, Fig. S12*B*). PB analysis demonstrated successful lymphoid reconstitution, with both T and B cells detected in primary and secondary recipients in both groups (*SI Appendix*, Figs. S12 *C* and *D* and S13). CBC analysis further demonstrated normal hematological parameters in secondary recipients in both groups, indicating sustained hematopoietic recovery following transplantation (*SI Appendix*, Fig. S12*E*). Notably, thymic reconstitution was observed in tertiary recipient at 4 wk posttransplantation (*SI Appendix*, Fig. S12 *F* and *G*). Together, these findings indicate that transient ATM inhibition enables correction of the *Il2rg* mutation while preserving the long-term repopulating capacity of genome-edited HSCs, resulting in sustained hematopoietic and immune reconstitution in the X-SCID mouse model.

### Transient ATM Inhibition Does Not Increase Unintended Genomic Alterations After Genome Editing.

**Fig. 6.**
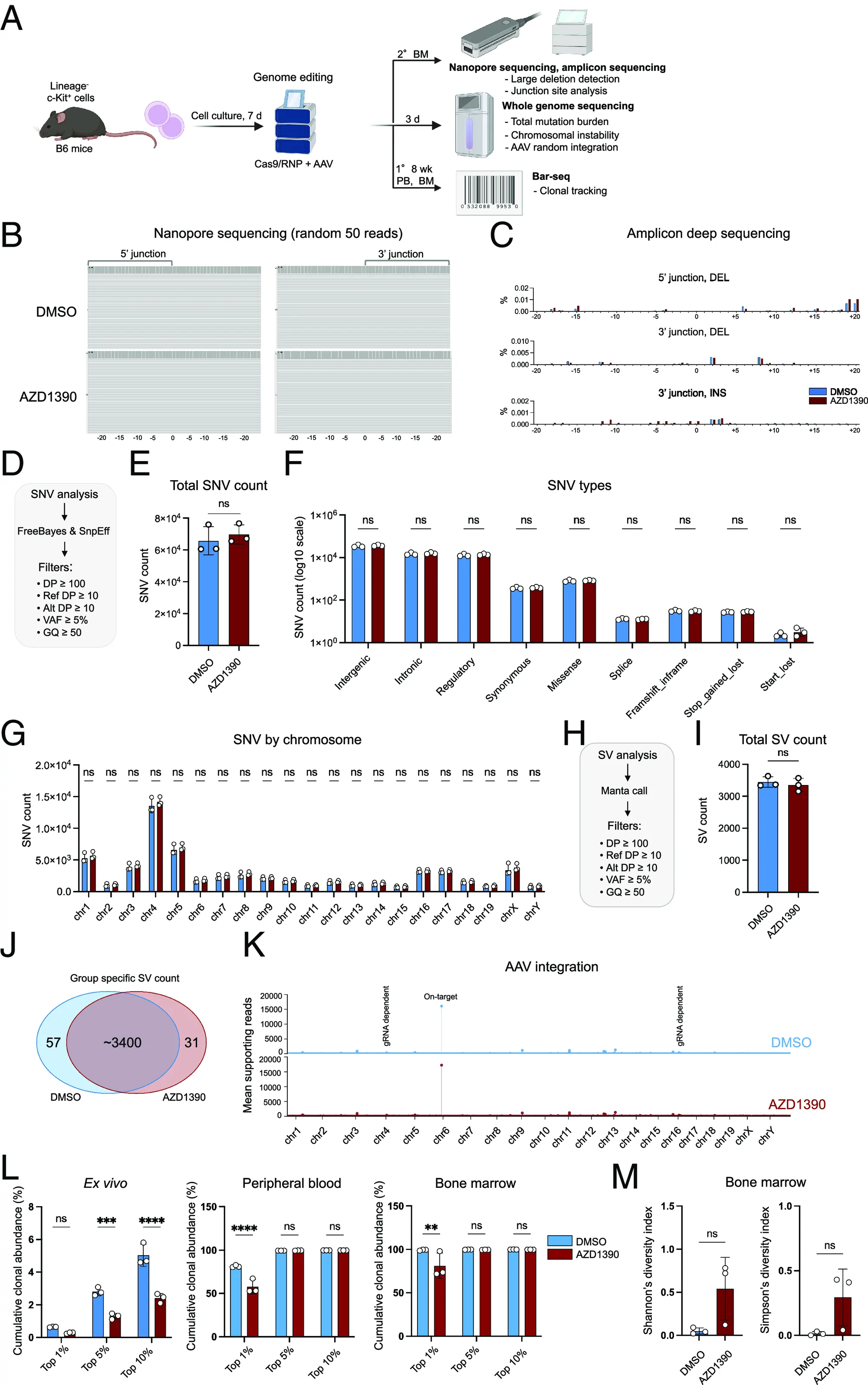
Genomic safety evaluation following transient ATM inhibition during genome editing. (*A*) Schematic of the experimental procedure for safety evaluation following genome editing. (*B*) Representative nanopore sequencing results showing randomly selected 50 bp-length KI reads for both the 5′ and 3′ junctions. No additional mutations were detected in the junction regions. (*C*) Amplicon deep sequencing results of the 5′ and 3′ junction sites. No increase in additional mutations was observed following AZD1390 treatment. (*D*) Schematic of the Single nucleotide variant (SNV) analysis workflow. SNVs were identified using FreeBayes and annotated using SnpEff. Variants were filtered using the following criteria: Sequencing depth (DP) ≥ 100, Reference depth (Ref DP) ≥ 10, Alternative depth (Alt DP) ≥ 10, Variant allele frequency (VAF) ≥ 5%, and genotype quality (GQ) ≥ 50. (*E*) Total SNV counts identified from WGS analysis after filtering. Data are presented as the mean ± SD (n = 3). Mann–Whitney test was conducted. *P* = 0.4. (*F*) Distribution of SNVs according to genomic annotation categories. Data are presented as the mean ± SD (n = 3). Šídák’s multiple comparisons test was conducted. (*G*) Distribution of SNVs across mouse chromosomes. Data are presented as the mean ± SD (n = 3). Šídák’s multiple comparisons test was conducted. (*H*) Schematic of the structural variant (SV) analysis workflow. SVs were identified using Manta. Variants were filtered using the following criteria: DP ≥ 100, Ref DP ≥ 10, Alt DP ≥ 10, VAF ≥ 5%, and GQ ≥ 50. (*I*) Total SV counts identified from WGS analysis after filtering. Data are presented as the mean ± SD (n = 3). Unpaired *t* test was conducted. *P* = 0.5960. (*J*) Venn diagram shows SVs between groups showing group-specific and shared SVs. (*K*) Lollipop diagram shows AAV integration sites throughout the genome. AAV integration events were predominantly detected at the on-target site. Two gRNA-dependent AAV integration events were detected, whereas the remaining integration events were gRNA independent. (*L*) Barcoded tracking results. Clonal abundance distributions were evaluated by calculating the cumulative contribution of the top 1, 5, and 10% most abundant barcode-defined clones. Ex vivo cultured cells showed a significantly lower cumulative abundance of the top 5% and top 10% clones in the AZD1390-treated group compared with DMSO controls, indicating a more evenly distributed clonal composition. Following transplantation, PB analysis demonstrated a reduced contribution of the top 1% clones in AZD1390-treated recipients, whereas no significant differences were observed for the top 5% or top 10% clones. In BM, AZD1390 treatment similarly reduced the contribution of the top 1% clones without affecting the top 5% or top 10% fractions. For ex vivo genome-edited cells, data are presented as the mean ± SD (n = 3). Šídák’s multiple comparisons test was conducted. DMSO vs. AZD1390, in top 1%, *P* = 0.4980; in top 5%, *P* = 0.0003; in top 10%, *P* < 0.0001. For PB of transplanted mice, data are presented as the mean ± SD (n = 3). Šídák’s multiple comparisons test was conducted. DMSO vs. AZD1390, in top 1%, *P* < 0.0001; in top 5%, *P* > 0.9999; in top 10%, *P* > 0.9999. For BM of transplanted mice, data are presented as the mean ± SD (n = 3). Šídák’s multiple comparisons test was conducted. DMSO vs. AZD1390, in top 1%, *P* < 0.0088; in top 5%, *P* > 0.9999; in top 10%, *P* > 0.9999. (*M*) Shannon’s and Simpson’s diversity indices were calculated from barcode abundance profiles of BM samples. Although both diversity indices tended to increase following AZD1390 treatment for 2 replicates, the differences did not reach statistical significance. For Shannon’s diversity index, data are presented as the mean ± SD (n = 3). Unpaired *t* test was conducted, *P* = 0.1443. For Simpson’s diversity index, data are presented as the mean ± SD (n = 3). Unpaired t test was conducted, *P* = 0.1573.

Because transient inhibition of DDR signaling could potentially affect genomic integrity, we next comprehensively evaluated the genomic safety of transient ATM inhibition after genome editing (Fig. 6*A*). First, we performed long-read nanopore sequencing to examine whether transient ATM inhibition increased large deletions (>41 bp by Dajin2) (DELs) surrounding the Cas9 target site. A 9.3-kb genomic region encompassing the genome-editing site was amplified from cells in the Cas9/RNP-only, Cas9/RNP+AAV+DMSO, and Cas9/RNP+AAV+AZD1390 groups 3 d after nucleofection (*SI Appendix*, Fig. S14*A*). Because amplification efficiency varies according to deletion size, absolute deletion frequencies could not be accurately quantified; therefore, relative deletion ratios were compared among groups. Comparable deletion ratios were observed in all three genome-edited groups (RNP: RNP+AAV+DMSO: RNP+AAV+AZD1390 = 1: 0.99: 0.94), indicating that transient ATM inhibition did not increase large deletion events (*SI Appendix*, Fig S14 *B* and *C*).

We next examined the integrity of full-length KI alleles using BM DNA from secondary recipients by the nanopore sequencing. Representative KI reads containing both the 5′ and 3′ junctions showed precise integration of the donor sequence without additional mutations at the junction sites (Fig. 6*B*). Consistent with these findings, amplicon deep sequencing of the KI junction regions did not reveal an increase in unintended mutations following AZD1390 treatment (Fig. 6 *C* and *D*).

To further evaluate genome-wide genetic alterations, whole-genome sequencing (WGS) was performed using genome-edited cells with or without AZD1390 treatment (Fig. 6 *D*–*K* and *SI Appendix*, Fig. S15). SNVs were identified using FreeBayes and annotated using SnpEff (Fig. 6*D*). After quality filtering, the total number of SNVs was comparable between the DMSO- and AZD1390-treated groups (Fig. 6*E*). Likewise, the distribution of SNVs across genomic annotation categories, including intergenic, intronic, regulatory, synonymous, missense, splice, and other variants, was similar between groups (Fig. 6*F*). Chromosome-level analysis also showed no apparent enrichment of SNVs in specific chromosomes in the AZD1390-treated group (Fig. 6*G*).

Structural variant (SV) analysis was performed using Manta (Fig. 6*H*). The total number of SVs did not differ substantially between the DMSO- and AZD1390-treated groups (Fig. 6*I* and *SI Appendix,* Fig. S15 *F* and *G*). Among these, several breakend (BND) events were identified as group-specific SVs in both groups (Fig. 6*J*). BAM reinspection showed that many were not supported as group-specific events, whereas a few BNDs retained group-specific junction support and others remained unresolved. As these candidates were not independently validated, definitive classification as true or false-positive SVs requires further analysis (*SI Appendix*, Table S1). CNV analysis similarly showed no detectable differences between the groups (*SI Appendix*, Fig. S15 *H*–*L*).

Because donor DNA delivery by AAV vector may result in vector integration throughout the genome, we next performed genome-wide AAV integration analysis using WGS data. The vast majority of AAV integration events were detected at the intended on-target locus, whereas only minimal gRNA independent AAV integration events were detected in both groups (Fig. 6*K*). 51 gRNA-independent AAV integration events were detected; however, there were no marked increase by AZD1390 treatment (*SI Appendix*, Fig. S16). Together, these findings indicate that transient ATM inhibition does not increase the frequency or genomic distribution of detectable AAV integration events.

Finally, to determine whether transient ATM inhibition influences the clonal composition of genome-edited HSCs, we performed clonal tracking using barcoded AAV donors. The plasmid and AAV barcode libraries exhibited high complexity, containing approximately 2.4 × 10^6^ and 5.5 × 10^5^ unique barcodes, respectively (*SI Appendix*, Fig. S17*A*). Both libraries also showed high Shannon’s and Simpson’s diversity, confirming sufficient barcode diversity for clonal tracking. Ex vivo, AZD1390-treated cells exhibited significantly reduced contributions from the top 5%, and top 10% most abundant clones compared with DMSO-treated controls, whereas the contribution of the single most abundant clone (top 1%) remained unchanged (Fig. 6*L*). These changes occurred without detectable alterations in overall diversity, as assessed by Shannon’s and Simpson’s diversity indices (*SI Appendix*, Fig. S17*B*). These findings indicate that AZD1390 treatment promotes a more even distribution of genome-edited clones immediately following genome editing.

Following transplantation, the cumulative abundance from the top 1% clones was significantly lower in the AZD1390-treated group in PB, whereas the cumulative abundance of the top 5% and top 10% clones remained comparable between groups. In BP, Shannon’s diversity index was comparable between groups, whereas Simpson’s diversity index was significantly higher in the AZD1390-treated group (*SI* *Appendix*, Fig. S17*C*). A similar pattern was observed in BM, where the cumulative abundance of the top 1% clones was significantly lower in the AZD1390-treated group, while the cumulative abundance from the top 5% and top 10% of clones remained comparable between groups (Fig. 6*L*). In BM, both Shannon’s and Simpson’s diversity indices were numerically higher in AZD1390-treated group, although neither difference reached statistical significance (Fig. 6*M*). Despite interrecipient variability, these results provide no evidence that transient ATM inhibition preferentially promotes the expansion of a restricted number of genome-edited HSC clones. Instead, they support the preservation of a highly polyclonal pattern of hematopoietic reconstitution within the period examined.

## Discussion

In this study, we demonstrate that transient inhibition of ATM enhances KI efficiency in mouse HSCs (CD201^+^ CD150^+^ KSL ex vivo and CD150^+^ CD48^−^ KSL in vivo). Mechanistically, this improvement was achieved by suppressing the DDR triggered by Cas9 and AAV donor delivery, which otherwise induced excessive ATM activation and subsequent p53-dependent apoptosis.

ATM is a key regulator of DDR and is one of the earliest sensors of DSB. Under conditions such as ionizing radiation (IR) or treatment with genotoxic agents, such as etoposide, multiple DSBs activate ATM, leading to apoptosis via p53 and caspase-3 signaling (25, 34, 35). Even a single unrepaired DSB can activate the apoptotic pathway (36). During genome editing, the CRISPR/Cas9 system typically generates targeted DSB (37). As an early sensor of DSB, appropriate ATM activation is required for DNA repair. Early ATM activation induces CHK2–p53–p21 signaling, resulting in G1 arrest that favors NHEJ (38). If a DSB is not repaired by NHEJ, HDR may occur, particularly during the S and G2 phases of the cell cycle, especially when plasmid donor DNA is used (39). Notably, genome editing with Cas9/RNP and AAV donor DNA appears to mimic multiple DSBs due to high AAV copy numbers, leading to ATM overactivation and apoptosis. Although higher amounts of donor DNA increase the likelihood of successful KI, they paradoxically induce cell death in KI-competent cells (40). Our findings demonstrate that ATM inhibition mitigates this detrimental overactivation of the apoptotic pathway, thereby preserving cells with high KI potential.

Phosphoproteomic analysis further revealed that AZD1390 treatment modulated early DDR signaling. Specifically, phosphorylation of canonical ATM substrates was reduced, whereas phosphorylation of NPM1 at Ser10 was increased. NPM1 is a multifunctional protein that is involved in genomic stability, chromatin structure regulation, and DNA repair (41). While NPM1 mutations in acute myeloid leukemia impair p53 signaling (42), wild-type NPM1 promotes cell cycle progression by regulating CDK1. Phosphorylation of NPM1 at Ser10 is associated with the G2/M transition and chromatin condensation, processes that are favorable for HDR (43). AZD1390 treatment also decreased CHK2 phosphorylation and p21 protein levels (*SI Appendix*, Fig. S8). Although p21 has been implicated in regulating DNA repair pathway choice, cell-cycle analysis at 4 and 16 h after nucleofection revealed no enrichment of cells in the HDR-permissive S/G2 phases following AZD1390 treatment. These findings indicate that the enhanced KI efficiency is unlikely to be primarily mediated by altered cell-cycle progression and instead reflects modulation of DNA damage signaling, apoptosis, and DNA repair pathway selection.

Consistent with previous studies in human HSPCs, transient inhibition of p53 using GSE56 also enhanced KI efficiency in mouse HSPCs (44). Previous studies have demonstrated that transient p53 inhibition improves KI efficiency in human HSPCs (45–47). However, p53 also plays a critical role in maintaining HSC quiescence and long-term stem cell function (48, 49). Although GSE56 significantly increased KI efficiency, AZD1390 produced greater preservation of KI HSCs without additional benefit from combined treatment. These findings indicate that attenuation of p53 signaling contributes substantially to the beneficial effects of ATM inhibition but does not fully account for them. Consistent with this interpretation, AZD1390 also suppressed phosphorylation of 53BP1, a downstream effector of ATM involved in DNA damage signaling and DSB repair. Although the contribution of 53BP1 to the enhanced KI efficiency remains to be determined, this observation suggests that transient ATM inhibition may modulate additional downstream pathways beyond p53, which could contribute to its superior effects compared with p53 inhibition alone. Together with the absence of additive effects between GSE56 and AZD1390, our results suggest that transient ATM inhibition acts predominantly through the p53 pathway, while additional p53-independent mechanisms are also likely to contribute.

HSCs are intrinsically quiescent and therefore typically favor NHEJ over HDR. To address this, we used ex vivo cultures to stimulate mouse HSC cycling and promote KI (50). After 7 d of culture, we obtained approximately 40% KI efficiency using Cas9/RNP and AAV donor DNA. However, without AZD1390 treatment, transplantation experiments showed a rapid loss of KI HSC engraftment, with only ~0.3% of genome-edited HSCs detectable in the BM 12 wk after secondary transplantation. This loss is most likely attributable to sustained DDR activation and subsequent apoptosis (51). Previous studies have reported similar findings: AAV transduction alone in human CD34^+^ HSPCs induced massive phosphorylation of H2AX, a key DDR marker (52), while AAV donor delivery activated the p53-related pathway and induced apoptosis in human mobilized PB (mPB) HSPCs (53). To overcome these barriers, prior strategies to enhance HDR in human HSCs have combined inhibition of NHEJ with suppression of p53. For example, delivery of mRNA for dominant negative truncated p53 (GSE56) together with transient p53 inhibition via Ad5-E4orf6/7 mRNA or GSE56+Ad5-E4orf6/7 mRNA+53BP1 inhibitor mRNA achieved ~50% KI efficiency in BM-derived CD34^+^ cells after secondary transplantation (45, 54). Similarly, the DNA-PKcs inhibitor AZD7648 combined with p53 siRNA increased KI efficiency to >90% in cord blood-derived CD34^+^ cells ex vivo (55). These approaches share two defining features: the inhibition of NHEJ and the suppression of p53, both of which are accomplished through transient delivery of RNA molecules. In contrast, our approach relies on transient pharmacological inhibition of ATM. This intervention suppressed p53 activation while reducing H2AX phosphorylation, thereby attenuating DDR signaling without requiring RNA- or peptide-based reagents. In our transplantation experiment, more than 40% of KI HSCs (mEGFP^+^ CD150^+^ CD48^−^ KSL) were preserved at 12 wk in secondary recipients in the AZD1390 group. These findings indicate that transient ATM inhibition not only enhances KI efficiency but also preserves multilineage repopulating HSCs by preventing stress-induced apoptosis. Notably, because ATM inhibition does not completely abolish NHEJ activity, it may strike a balance between promoting KI and preserving essential repair capacity in cases where KI does not successfully resolve the break.

To assess the therapeutic applicability of our approach, we used a mouse model of X-SCID caused by a frameshift mutation in *Il2rg*. Because corrected lymphoid cells gain a strong selective advantage, even relatively low levels of gene correction may contribute to immune reconstitution (56). Consistent with the findings obtained using the mEGFP reporter system, AZD1390 significantly enhanced KI efficiency during *Il2rg* correction in mouse HSPCs. Following serial transplantation, corrected HSCs contributed to durable hematopoiesis and sustained T cell, B cell, and thymic reconstitution. These findings demonstrate functional correction of the *Il2rg* mutation and support its potential application of this approach for the treatment of genetic hematological disorders.

An important concern regarding transient suppression of DDR signaling is the potential induction of genomic instability. Our strategy, however, was designed to transiently attenuate the excessive ATM-dependent DDR induced by AAV donor delivery, rather than abolish ATM function or broadly suppress DNA repair. Nevertheless, given the central role of ATM in maintaining genomic integrity, we evaluated genomic integrity at the local, genome-wide, and clonal levels. At the target locus, long-read nanopore sequencing and amplicon deep sequencing showed no increase in large deletions or unintended alterations at the KI junctions following AZD1390 treatment, and analysis of BM DNA from secondary recipients confirmed intact donor integration (Fig. 6*C* and *SI* *Appendix*, Fig. S14 *B–D*). Furthermore, WGS revealed comparable numbers and distributions of SNVs, SVs, and CNVs between DMSO- and AZD1390-treated groups. Reinspection of candidate group-specific SVs showed that many were not supported as group-specific events, whereas a few BNDs retained group-specific junction support and others remained unresolved, warranting further validation. Genome-wide AAV integration analysis likewise showed no increase or apparent redistribution of candidate gRNA-dependent or gRNA-independent integration events (Fig. 6*K*). Barcoded clonal tracking further provided no evidence that transient ATM inhibition promoted clonal dominance or preferential expansion of a restricted number of genome-edited HSC clones. Instead, AZD1390 reduced clonal skewing while maintaining overall clonal diversity and polyclonal hematopoietic contribution. Collectively, these complementary analyses provide no evidence that transient ATM inhibition increases genomic instability, chromosomal abnormalities, random AAV integration, or clonal dominance under the conditions examined in this study.

Notably, AZD1390 is currently being evaluated clinically for glioblastoma (57), the translational feasibility of pharmacological ATM inhibition, although further long-term evaluation will be required to establish its safety in genome-edited HSCs.

In conclusion, our study demonstrates that transient ATM inhibition strikingly enhances KI efficiency and preserves long-term repopulating KI HSCs and enables effective functional correction in disease models. Importantly, extensive genomic analyses did not reveal increased genomic instability, genome-wide AAV integration, or clonal dominance following transient ATM inhibition. Together, these findings establish transient pharmacological inhibition of excessive ATM activation during AAV-mediated genome editing as a simple, broadly applicable, and potentially translatable strategy for improving precise genome editing in HSCs.

## Materials and Methods

Detailed *Materials and Methods* are provided in the *SI Appendix*.

### Mice.

C57BL/6J (CD45.2) mice (Japan SLC, Inc), NOD.Cg-*Prkdc^scid^Il2rg^tm1Sug^*/ShiJic (NOG) mice (In Vivo Science Inc) and C57BL/6J (CD45.1) mice (kindly provided by Dr. Atsushi Iwama) were used for genome editing and transplantation assays. Previously generated X-SCID mice were used as disease model (58). All animal studies were conducted in accordance with the ARRIVE guidelines and were approved by the Institutional Animal Care and Concern Committee at Jichi Medical University. All efforts were made to ensure the animal welfare and minimize pain and distress during the experimental procedures.

### Cell Culture.

Mouse 32D cells and Hes1-CMP (59) cells were cultured in cytokine supplemented RPMI-1640 or IMDM medium, respectively, at 37 °C under 5% CO_2_ conditions. Ex vivo isolated LK cells from C57BL/6 J mice were cultured in HemEx-Type 9A medium supplemented with mTPO, mSCF, HEPES, ITS-X, and penicillin–streptomycin. LK cells were maintained on fibronectin-coated plates with medium replacement every 2 d.

### Genome Editing Procedures.

Cas9/RNP complexes were assembled by combining Cas9 protein with annealed tracrRNA and target-specific crRNA (60). AAV donor vectors were produced, purified, and quantified as described previously (61, 62). Genome editing was performed by nucleofection of Cas9/RNP, immediately followed by AAV donor transduction. ATM inhibitors or vehicle control (DMSO) were added immediately after genome editing, whereas GSE56 (32) was included in experiments evaluating p53 inhibition. KI efficiency was determined by flow cytometry for mEGFP KI experiments or by Sanger sequencing with ICE analysis for X-SCID experiments.

### Serial BM Transplantation in Mice.

Genome-edited LK cells were transplanted into lethally irradiated recipient mice together with accessory BM cells, followed by serial transplantation into secondary recipients. PB and BM samples were collected after transplantation to evaluate long-term hematopoietic reconstitution and stem cell engraftment. For X-SCID experiments, genome-edited LK cells were transplanted into sublethally irradiated NOG mice and serially transplanted using the same strategy. Donor engraftment and immune reconstitution were evaluated by flow cytometry.

### Capillary Western Blotting.

Ex vivo cultured LK cells were genome-edited using Cas9/RNP and AAV donor DNA and harvested 3 to 4 h postnucleofection for protein extraction. Cell lysates were prepared and protein concentrations were quantified before analysis. Capillary western blotting was performed using the JESS system (Bio-Techne) according to the manufacturer’s instructions.

### Amplicon Deep Sequencing.

gDNA isolated from the BM of secondary recipient mice was used for knock-in junction deep sequencing. Junction-spanning PCR followed by nested PCR was performed to eliminate potential contamination from residual AAV donor DNA and generate sequencing libraries. Amplicons were sequenced on a MiSeq i100 platform (Illumina). Sequencing data were analyzed using CRISPResso2 (63).

### WGS.

gDNA was extracted from genome-edited LK cells 72 h postnucleofection and subjected to PCR-free WGS on the NovaSeq X Plus platform (Illumina). Sequence reads were processed and aligned to the mouse reference genome (GRCm39/mm39), and single-nucleotide variants, structural variants, and copy number variations were identified using established bioinformatic pipelines (64–70). Candidate structural variants were further evaluated by manual inspection of paired-end and split-read evidence. Genome-wide donor integration events were analyzed using a custom computational pipeline (available at https://github.com/Ohmori-Insilico/AAV-Donor-Integration-Pipeline), which identified donor-supporting alignments, clustered candidate integration loci, and classified integration events as on-target, gRNA-dependent off-target, or gRNA-independent.

### Statistical Analysis.

Statistical analyses were performed using GraphPad Prism 10. Data are presented as mean ± SD, and percentage values were arcsine transformed before analysis. Appropriate parametric or nonparametric statistical tests were applied according to data distribution. Statistical methods used for each experiment are indicated in the corresponding figure legends.

## Supplementary Material

Appendix 01 (PDF)

## Data Availability

The NGS data generated in this study, including WGS and other sequencing datasets, have been deposited in the NCBI BioProject database under accession PRJNA1538659 (71). The phosphoproteomics data have been deposited in the MassIVE repository under accession MSV000103462 (72) and ProteomeXchange under accession PXD085129 (73). Additional source data, including image and Sanger sequencing files, have been deposited in Zenodo under DOI 10.5281/zenodo.23131349 (74). All deposited data will be publicly accessible upon publication. All other data are included in the article and/or *SI Appendix*.
