# Supplementary material for: Transient ATM inhibition enhances knock-in efficiency in hematopoietic stem cells by attenuating the DNA damage response
Source: Proc Natl Acad Sci U S A. 2026 Oct 20;123(43):e2531364123. doi: 10.1073/pnas.2531364123 (PMC13715756; doi:10.1073/pnas.2531364123)
Supplement: Supplementary file 1 [file pnas.2531364123.sapp.pdf]

Transient ATM inhibition enhances knock-in efficiency in hematopoietic stem cells by attenuating the DNA damage response

Munkh-Erdene Natsagdorj[1,†], Hiromasa Hara[1-4†], Kohei Iida[1], Yuji Kashiwakura[5], Nemekhbayar Baatartsogt[5], Enkhzaya Ganzorig[1], Daniele Canarutto[6,7], Sandra Pinho[8], Luigi Naldini[6,7], Tsukasa Ohmori[5], Yutaka Hanazono[1,2,9], Fumio Nakahara[1]

[1]Division of Regenerative Medicine, Center for Molecular Medicine, Jichi Medical University, Shimotsuke, Tochigi, Japan; [2]Laboratory of Regenerative and Cellular Medicine, Center for Development of Advanced Medical Technology, Jichi Medical University, 3311-1 Yakushiji, Shimotsuke, Tochigi 329-0498, Japan; [3]Division of Animal Resource Development, Center for Development of Advanced Medical Technology, Jichi Medical University, 3311-1 Yakushiji, Shimotsuke, Tochigi 329-0498, Japan; [4]Center for Experimental Medicine, Jichi Medical University, 3311-1 Yakushiji, Shimotsuke, Tochigi 329-0498, Japan; [5]Department of Biochemistry, School of Medicine, Jichi Medical University, Shimotsuke, Tochigi, Japan; [6]San Raffaele Telethon Institute for Gene Therapy, IRCCS San Raffaele Scientific Institute, Milan 20132, Italy; [7]Vita-Salute San Raffaele University, Milan 20132, Italy; [8]Department of Pharmacology & Regenerative Medicine; University of Illinois at Chicago, Chicago, USA; [9]Translational Research Laboratory, Center for Development of Advanced Medical Technology, Jichi Medical University, 3311-1 Yakushiji, Shimotsuke, Tochigi 329-0498, Japan

Correspondence: Fumio Nakahara

Extended Materials and Methods

Mice

C57BL/6J (CD45.2) mice (Japan SLC,Inc), NOD.Cg-*Prkdc^scid^Il2rg^tm1Sug^*/ShiJic (NOG) mice (In-Vivo Science Inc) and C57BL/6J (CD45.1) mice (kindly provided by Dr. Atsushi Iwama) were used for genome editing and transplantation assays. Previously generated X-SCID mice were used as disease model (1). All animal studies were conducted in accordance with the ARRIVE guidelines and were approved by the Institutional Animal Care and Concern Committee at Jichi Medical University. All efforts were made to ensure the animal welfare and minimize pain and distress during the experimental procedures.

Preparation of mouse Lineage$^-$ and c-Kit$^+$ cells

To obtain bone marrow (BM) cells were harvested from tibias and femurs of 8-week-old mice. Red blood cells were removed with 1×RBC lysis buffer (Pluriselect). Lineage$^-$ and c-Kit$^+$ (LK) cells were isolated using Direct Lineage Depletion cocktail and mouse CD117 microbeads (Miltenyi Biotech, Germany) on autoMACS separator.

Cell culture

Mouse 32D cells were cultured in RPMI-1640 medium (Gibco) supplemented with 10% Fetal Bovine Serum (Gibco), 1 µg/ml Mouse IL-3 (Peprotech), 1 × Glutamax (Gibco) and 1 × penicillin streptomycin (Gibco). Cells were maintained at 37°C in a humidified atmosphere with 5% CO2, and medium was changed every 2-3 days. Hes1-CMP cells (2) were cultured in IMDM medium (Gibco) supplemented with 10% Fetal Bovine Serum (Gibco), 1 µg/ml Mouse IL-3 (Peprotech), 1 × Glutamax (Gibco) and 1 × P/S (Gibco). The culture conditions and medium replacement schedule were the same for 32D.

Ex vivo-isolated LK cells from C57BL/6J mice were cultured in HemEx-Type 9A (Cell Science & Technology Institute, Inc) supplemented with 100 ng/ml Mouse TPO (Peprotech), 10 ng/ml Mouse SCF (Peprotech), 10 µM HEPES (Gibco), 1 × Insulin-Transferrin-Selenium-Ethanolamine (ITS-X) (Gibco) and 1 × penicillin–streptomycin (P/S) (Gibco). Cells were seeded in fibronectin-coated plates (Corning) and maintained at 37°C in a 5% CO_2, atmosphere, with medium changed every 2 days.

Genome editing procedures

To prepare the Cas9/RNP, tracrRNA and target-specific crRNA (both from IDT) were mixed, heated at 95°C for 5 minutes, and then cooled 25°C to allow duplex formation. The duplex was then combined with Cas9 protein (IDT) to a final concentration of 18.6 µM and incubated at room temperature for 20 minutes to assemble the Cas9/RNP complex (3).

AAV vectors were used as donor DNA for genome editing. The recombinant expression cassette was packaged into AAV capsids by triple-plasmid transfection of AAVpro293T cells (Takara Bio). AAV vectors were purified from the transfected cells by ultracentrifugation, as described previously (4, 5). Vector titers were determined by quantitative PCR (qPCR) of the SV40 polyadenylation signal. The qPCR primer and probe were as follows: forward, GCAATAGCATCACAAATTTCAC; reverse, GATCCAGACATGATAAGATACATTG; and probe, TCACTGCATTCTAGTTGTGGTTTGTCCA.

For delivery of the Cas9/RNP (final concentration: 0.744 µM), the Lonza 4D-Nucleofector™ system was used. The CM-137 electroporation program was applied for mouse LK cells, 32D cells and Hes1-CMP cells. Immediately after nucleofection, cells were transduced with AAV donor DNA at 1 × 10^4 viral genomes (vg)/cell. The culture medium was replaced 24 h post-nucleofection to remove residual AAV particles. To enhance KI efficiency, ATM inhibitors were added to the culture medium immediately after genome editing. For vehicle control, DMSO was added at a concentration matched to the ATM inhibitor condition (0.0001% v/v). The medium was changed after 24 h to remove any compounds. A list of ATM inhibitors is provided in *SI appendix*, Table S2. To test the effect of p53 inhibition, GSE56 (6) at 150 µg/ml concentration were added to the nucleofection mix. Following treatments, cells were cultured for an additional 2–5 days before analysis.

For mEGFP KI experiments, KI efficiency was assessed by flow cytometry (Sony SH800) based on the percentage of mEGFP$^+$ cells among total live cells or within specific fractions, such as CD201$^+$ CD150$^+$ c-Kit$^+$ Sca1$^+$ Lineage$^-$ cells. All antibodies for flow cytometry are listed in *SI appendix*, Table S3.

For X-SCID mouse treatment experiments, KI efficiency for Interleukin-2 receptor subunit gamma (*Il2rg*) correction was evaluated via Sanger sequencing of PCR amplicons derived from genome-edited cells. Sequencing traces were analyzed using the ICE CRISPR Analysis online tool (v3.0, 2025; EditCo Bio) to quantify editing outcomes. The primers used for determining KI and KI efficiency are: F1: ACCAGACATTTGTTGTCCAG; F2: TTCCTAGAGCCCCTGAGAACC; R1: CTGACGTGGCAGAACCGT.

Serial BM transplantation in mice

For mEGFP KI experiments, a total of 2 × 10^5 genome-edited LK cells were cultured for 3 days and transplanted into lethally irradiated (9.5 Gy) C57BL/6J (CD45.2) mice via retro-orbital vein injection. As accessory cells, 1 × 10^5 BM cells from C57BL/6J (CD45.2) mice were co-transplanted. Twelve weeks after primary transplantation, 5 × 10^6 BM cells from primary recipient were transplanted into lethally irradiated (9.5 Gy) secondary C57BL/6J (CD45.2) mice. PB was analyzed 8 weeks after transplantation. Approximately 100 µl of PB was collected from recipient mice via submandibular vein puncture into ETDA-coated collection tube. Complete blood counts were performed using Celltac α automated hematology analyzer. For flow cytometry analysis, PB samples were centrifuged to separate plasma, and erythrocytes were lysed by 1 × RBC lysis buffer (Pluriselect). Then washed WBC were subsequently used for flow cytometry analysis on a BD LSRFortessa™ X-20 instrument. BM cells were analyzed 12 weeks post-transplantation. BM was harvested using the method described above. Following RBC lysis, $Lineage^-$ cells were isolated using AutoMACS in Depletes mode. The enriched cells were used for flow cytometry analysis, with $CD150^+$ $CD48^-$ $cKit^+$ $Sca1^+$ $Lineage^-$ cells defined as HSCs.

For X-SCID mouse treatment experiments, a total of 2 × 10^5 genome-edited LK cells from X-SCID mice, were cultured for 3 days and transplanted into lethally irradiated (2.5 Gy) NOG (CD45.1) mice via retro-orbital vein injection. As accessory cells, 1 × 10^5 BM cells from NOG mice were co-transplanted. Eight weeks after primary transplantation, 5 × 10^6 BM cells from primary recipient were transplanted into lethally irradiated (2.5 Gy) secondary NOG mice. PB and BM cells were analyzed by BD LSRFortessa™ after 8 weeks of the transplantations.

Phosphoproteomics analysis

LK cells were cultured *ex vivo* for 7 days and genome-edited using Cas9/RNP and AAV donor DNA. Cells were collected at 2, 6, or 24 h post-nucleofection, washed three times with ice-cold PBS, centrifuged to form a cell pellet, snap-frozen in liquid nitrogen, and stored at −80° C until further processing. Phosphoproteomic analysis, including sample preparation, phosphopeptide enrichment, LC-MS/MS acquisition, and data processing, was outsourced to BGI Genomics and conducted according to the provider's standard protocols. Peptide identification was performed using Proteome Discoverer 1.4 (Thermo Fisher Scientific) integrated with MASCOT 2.3 (7) for database searching. Percolator was used to reprocess the Mascot search results, and peptides were filtered using a false discovery rate threshold of ≤ 1.0% at the spectral level. Phosphorylation site localization was assessed using the phosphoRS module in Proteome Discoverer, with phosphosites considered confidently when the phosphoRS probability was ≥ 0.75 (8). Functional annotation and motif enrichment analyses were performed on proteins containing confidently localized phosphosites.

Capillary western blotting

LK cells were cultured *ex vivo* for 7 days and genome edited using Cas9/RNP and AAV donor DNA under above mentioned conditions. Cells were collected at 3-4 h post-nucleofection, washed with ice-cold PBS, and centrifuged to form a pellet. The pellets were lysed using M-PER (Thermo Fisher Scientific) supplemented with protease inhibitors (Nacalai Tesque) and a proteinase inhibitor cocktail (Abcam). Extracted proteins were mixed with 2 × Laemmli buffer (Bio-Rad) containing dithiothreitol at a final concentration of 100 mM and boiled at 95° C for 5 min. Protein concentration was measured using the Pierce™ 660 nm Protein Assay (Thermo Fisher Scientific). Capillary western blotting was performed using the JESS system (Bio-techne). The 66-440 kDa Fluorescence Separation Module was used for ATM and p-ATM; the 12-230 kDa Fluorescence Separation Module was used for GAPDH, NPM1, p-NPM1, p53, p-p53, CHK2 and p-CHK2, and the 2-40kDa Fluorescence Separation Module was used for p-H2AX, p21, Caspase 3 and Cleaved Caspase 3, according to the manufacturer's protocol. A complete list of antibodies is provided in *SI appendix*, Table S4.

Cell cycle analysis

LK cells were cultured for 7 days and genome edited using Cas9/RNP and AAV donor DNA with or without AZD1390 treatment. Cells were collected at 4 and 16h post-nucleofection, washed with PBS and stained with Zombie NIR™ (Biolegend) for dead cell exclusion. Cells were then fixed with BD Phosflow™ Fix Buffer I (BD Biosciences) and permeabilized with BD Phosflow™ Perm Buffer III (BD Biosciences). The DNA content was assessed by DAPI (Dojindo) staining. Data acquisition was performed on a SH800 (Sony) cell sorter. Cell cycle distribution was analyzed by Dean-Jett-Fox model using FlowJo™ 10 software.

Flow cytometry with Cellview™

Seven-day cultured LK cells were genome edited using Cas9/RNP and AAV donor DNA. Cells were collected at 3 h post-nucleofection, washed with PBS, and stained with antibodies for 30 minutes at 4°C. Surface markers included lineage markers (CD3, B220, Gr1, CD11b, Ter119), c-Kit, Sca-1, CD150 to define hematopoietic stem and progenitor cell populations. For intracellular staining (γH2AX), cells were fixed and permeabilized with BD Phosflow™ Fix Buffer I and BD Phosflow™ Perm Buffer III (BD Biosciences). Data acquisition was performed on a BD FACSDiscover™ S8 Cell Sorter with BD CellView™ Image Technology and BD SpectralFX™ Technology (BD Biosciences). Gates were defined using fluorescence minus one or isotype controls, where appropriate. Data were analyzed using FlowJo™ 10 software.

Long-read nanopore sequencing

Genomic DNA (gDNA) was extracted from genome-edited cells at 3 days post-nucleofection or from BM cells collected from secondary recipient mice using the DNeasy Blood & Tissue Kit (Qiagen). Long-range PCR was performed to amplify the *Actb* KI locus, generating an approximately 10 kb amplicon in KI alleles and a 9.3 kb amplicon in unedited alleles. Long-read amplicon sequencing, including the sample preparation was outsourced to the NGS Core Facility at the Research Institute for Microbial Diseases of The University of Osaka. Libraries were prepared using the Native Barcoding Kit 24 (SQK-NBD114-24, Oxford Nanopore Technologies) according to the manufacturer's instructions. Barcoded libraries were loaded onto FLO-MIN114 flow cells and sequenced on a MinION device (Oxford Nanopore Technologies). Base calling was performed using Dorado (v1.0.0) in super-accuracy mode. Sequencing reads were aligned to the reference amplicon sequence, and the Cas9 cleavage site and KI junction regions were analyzed to assess precise integration events and sequence alterations. The sequencing data were analyzed using Dajin2 and the 5′ and 3′ KI junctions were inspected using Samtools and IGV to assess precise donor integration and detect sequence alterations.

Amplicon deep sequencing

gDNA isolated from the BM of secondary recipients was used for junction deep sequencing analysis. To exclude potential contamination from residual AAV donor DNA, approximately 1.2-kb amplicons spanning the KI junctions were first amplified using a primer located upstream of the 5′ homology arm and a primer within the mEGFP sequence for the 5′ junction, or a primer within the mEGFP sequence and a primer located downstream of the 3′ homology arm for the 3′ junction. Subsequently, nested PCR was performed to amplify approximately 150-bp regions encompassing each junction site. The resulting amplicons were subjected to Illumina deep sequencing using the MiSeq i100 platform. Sequencing data were analyzed using CRISPResso2 (v2.3.3) (9).

Whole-genome sequencing

WGS gDNA was extracted from genome-edited LK cells at 72 h post-nucleofection using the DNeasy Blood & Tissue Kit (Qiagen). WGS analysis, including sample preparation was outsourced to Rhelixa, Inc. PCR-free WGS libraries were prepared using the NEBNext Ultra II DNA Library Prep Kit (NEB) and sequenced on a NovaSeq X Plus platform (Illumina) to generate 150-bp paired-end reads. Approximately 300 Gb of sequencing data were obtained for each sample. Sequence reads were quality-filtered using FastQC (v0.11.7) (10) and Trimmomatic (v0.38) (11),aligned to the mouse reference genome (GRCm39/mm39) using BWA-MEM (v0.7.17) (12), and duplicate reads were removed using Picard (v2.18.11) (http://broadinstitute.github.io/picard/. SNVs and small insertions/deletions were identified using FreeBayes (v1.3.4) (13) and annotated using SnpEff (v4.3t) (14). SVs were detected using Manta (v1.6.0) (15) and copy number variation analysis was performed using CNVkit (v0.9.8) (16). For downstream analyses, high-confidence SNVs were defined as variants with sequencing depth ≥100, alternative allele depth ≥10, variant allele frequency ≥5%, genotype quality ≥50, and a non-reference genotype. Group-specific variants were identified as variants consistently detected in all biological replicates of one experimental group and absent from the other group. SVs, including DEL, INS, DUP, INV, and BND, were identified using Manta. Supporting evidence for each SV was calculated as the sum of paired-read and split-read support. High-confidence SVs were defined as variants supported by at least 10 reads, whereas stringent analyses used a threshold of 20 supporting reads.

The filtered Manta SV calls (DP ≥100, reference and alternative depth ≥10, VAF ≥5%, and GQ ≥50) were analyzed using custom Python scripts. Variants with combined alternative paired-read and split-read support (PRALT + SRALT) <10 were excluded. Calls of the same SV type within 100 bp were clustered, and clusters represented by three records from one group and none from the other were classified as group-specific in the original analysis. This identified 57 Group 1- and 31 Group 2-specific SV calls (88 total): 36 BNDs, 40 INSs, 9 DELs, and 3 DUPs. The 88 calls were subsequently reassessed using BAM files from all six samples. For BNDs, reads at both breakpoints were examined for supplementary-alignment or mate-pair evidence linking the expected junctions within ±20 bp. DELs were evaluated using breakpoint-spanning CIGAR deletions, supplementary alignments, and paired-read evidence, whereas DUPs were assessed using supplementary alignments and abnormal paired-read evidence at the predicted breakpoints.

For INSs, Manta-derived insertion sequences (ALT, LEFT_SVINSSEQ, or RIGHT_SVINSSEQ) were searched in reads within ±150 bp of the predicted breakpoint, including soft-clipped and supplementary reads. Evidence was compared across both groups to assess whether the original group-specific designation was supported. These analyses represent bioinformatic reinspection of the WGS data rather than independent experimental validation; definitive classification of individual SVs as true or false positives would require orthogonal validation.

An unbiased genome-wide donor integration analysis was performed using a custom computational pipeline developed for this study (available at https://github.com/Ohmori-Insilico/AAV-Donor-Integration-Pipeline). Genome-wide soft-clipped and supplementary alignment signals supporting genome-donor was identified from the duplicate-marked BAM files. Donor-supporting reads were clustered into candidate loci based on genomic breakpoint proximity and quantified separately for each biological replicates (DMSO n = 3, AZD1390 n=3). Candidate loci were classified as: on-target integration, gRNA-dependent off-target integration (donor- junctions located near predicted Cas9 off-target sites), and gRNA-independent donor-junctions lacking nearby predicted gRNA sequence similarity and visualized as lollipop plots generated in R (v4.6.0).

Clonal tracking

To generate barcoded AAV donor vectors, a 22 bp barcode sequence (NNYNNNTNTNNNNRTNDNNNHH)(17) was introduced downstream of the mEGFP cassette. The barcode library was transformed into NEB 10-beta Electrocompetent *E.coli* (C3020K, NEB) using an ELEPO21 electroporator (Nepagene) using following conditions: poring pulse, 2,000 V for 2.5 ms with one pulse, and transfer pulse, 150 V for 50 ms with five pulses. Recombinant AAV vectors carrying the barcode library were subsequently produced as described above. Genome editing of LK cells was performed using Cas9/RNP and barcoded AAV donor vectors under the same conditions described for mEGFP KI experiments. Genome-edited cells were transplanted into recipient mice, and PB and BM samples were collected 8 weeks after transplantation. gDNA was extracted from the collected cells, and the barcode-containing region was amplified by PCR. Sequencing libraries were prepared from barcode amplicons and sequenced on NovaSeqX plus or MiSeq i100 platform (Illumina). Barcode sequencing data were analyzed using the BAR-Seq pipeline (https://bitbucket.org/bereste/bar-seq) (18).

Statistical analysis

Statistical analyses were performed using GraphPad Prism 10. Data are presented as mean ± standard deviation. Percentage values were arcsine-transformed prior to analysis, and normality was assessed using the Shapiro–Wilk test. Depending on distribution, parametric or nonparametric tests were applied, including Unpaired t-tests, Mann–Whitney tests, Tukey's multiple comparison tests, Dunn's multiple comparisons test and Šidák multiple comparison tests. All tests were two-sided with a 95% confidence interval, and $p < 0.05$ were considered statistically significant. Experiments were conducted in biological triplicate unless otherwise specified. Transplantation assays were performed with six mice per group. The specific statistical tests applied to each dataset are detailed in the figure legends.

Figure S1.

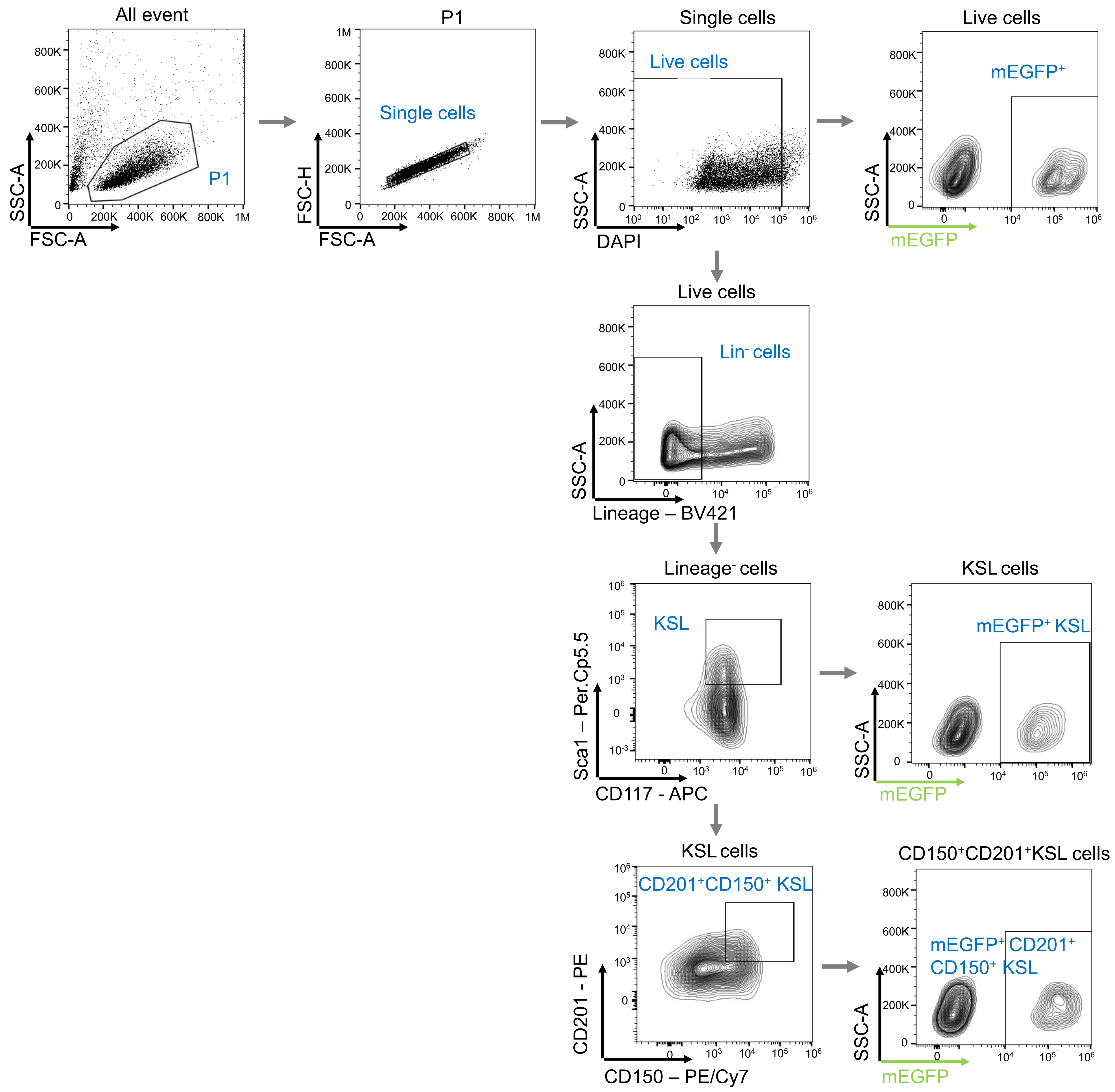

Gating strategy for flow cytometry analysis to evaluate KI efficiency of *ex vivo* genome editing of mouse HSC.

Figure S2.

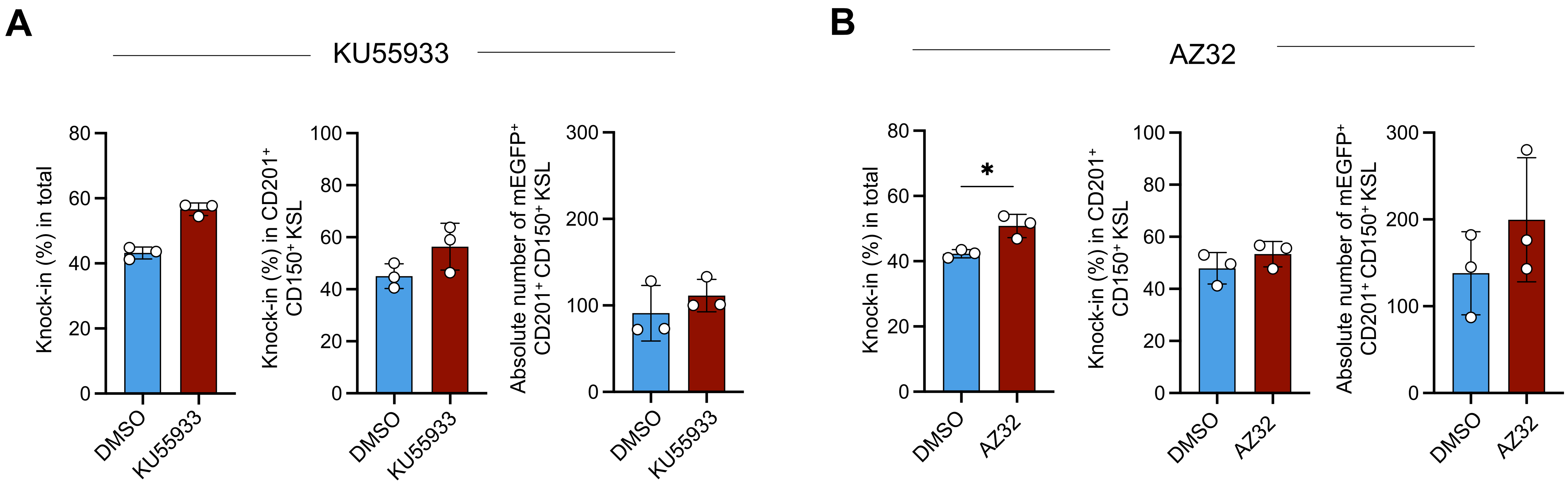

Some of the ATM inhibitors tested for genome editing in mouse HSCs. (A) Genome editing results following treatment with 1 μM KU55933. Data are presented as the mean ± standard deviation (SD) (n=3). KI (%) in total, Mann-Whitney t-test was conducted. $P = 0.100$; For KI (%) in HSCs, Unpaired t-test was conducted. $P = 0.1244$; For Absolute number of $mEGFP^+$ HSCs, Mann-Whitney t-test was conducted. $P = 0.400$. (B) Genome editing results following treatment with 1 μM AZ32. Data are presented as the mean ± standard deviation (SD) (n=3). KI (%) in total, Unpaired t-test was conducted. $P = 0.0169$; For KI (%) in HSCs, Unpaired t-test was conducted. $P = 0.2969$; For Absolute number of $mEGFP^+$ HSCs, Mann-Whitney t-test was conducted. $P = 0.2824$.

Figure S3.

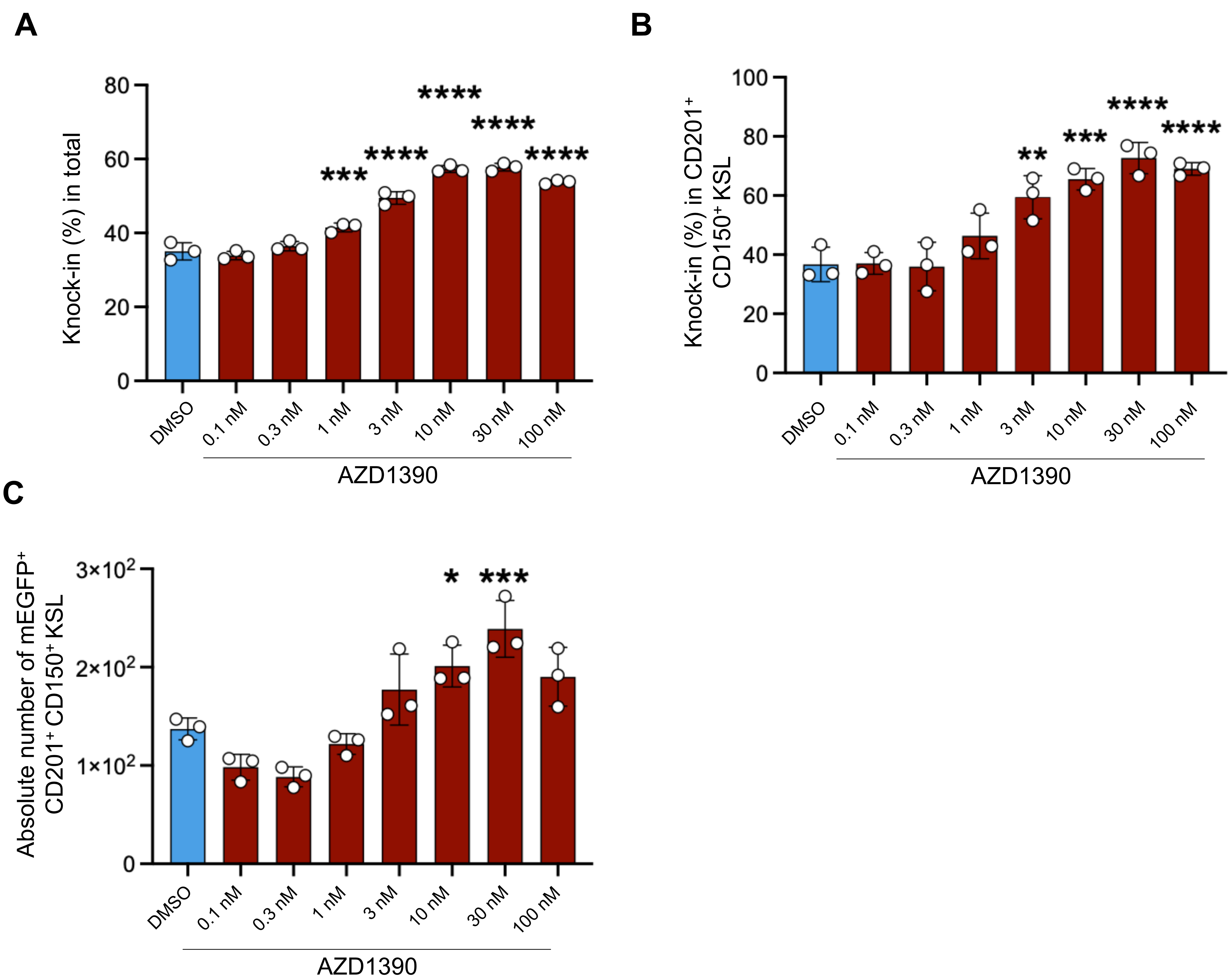

AZD1390 dose-dependently increased the knock-in (KI) efficiencies in HSPC and HSC fraction. (A) Total KI efficiencies following treatment with AZD1390. Data are presented as the mean ± standard deviation (SD) (n=3). Dunnett's multiple comparison test was conducted. NC vs 0.1 nM, $P = 0.8393$; NC vs 0.3 nM, $P = 0.6641$; NC vs 1 nM, $P = 0.0002$; NC vs 3 to 100 nM, $P < 0.0001$. (B) KI efficiencies in HSCs following treatment with AZD1390. Data are presented as the mean ± SD (n=3). Dunnett's multiple comparison test was conducted. NC vs 0.1 nM, $P > 0.9999$; NC vs 0.3 nM, $P > 0.9999$; NC vs 1 nM, $P = 0.2692$; NC vs 3 nM, $P = 0.0016$; NC vs 10 nM, $P = 0.0001$; NC vs 30 nM, $P < 0.0001$; NC vs 100 nM, $P < 0.0001$. (C) Absolute number of $mEGFP^+$ HSCs following treatment with AZD1390. Data are presented as the mean ± SD (n=3). Dunnett's multiple comparison test was conducted. NC vs 0.1 nM, $P = 0.2105$; NC vs 0.3 nM, $P = 0.0808$; NC vs 1 nM, $P = 0.9244$; NC vs 3 nM, $P = 0.1905$; NC vs 10 nM, $P = 0.0159$; NC vs 30 nM, $P = 0.0003$; NC vs 100 nM, $P = 0.0518$.

Figure S4.

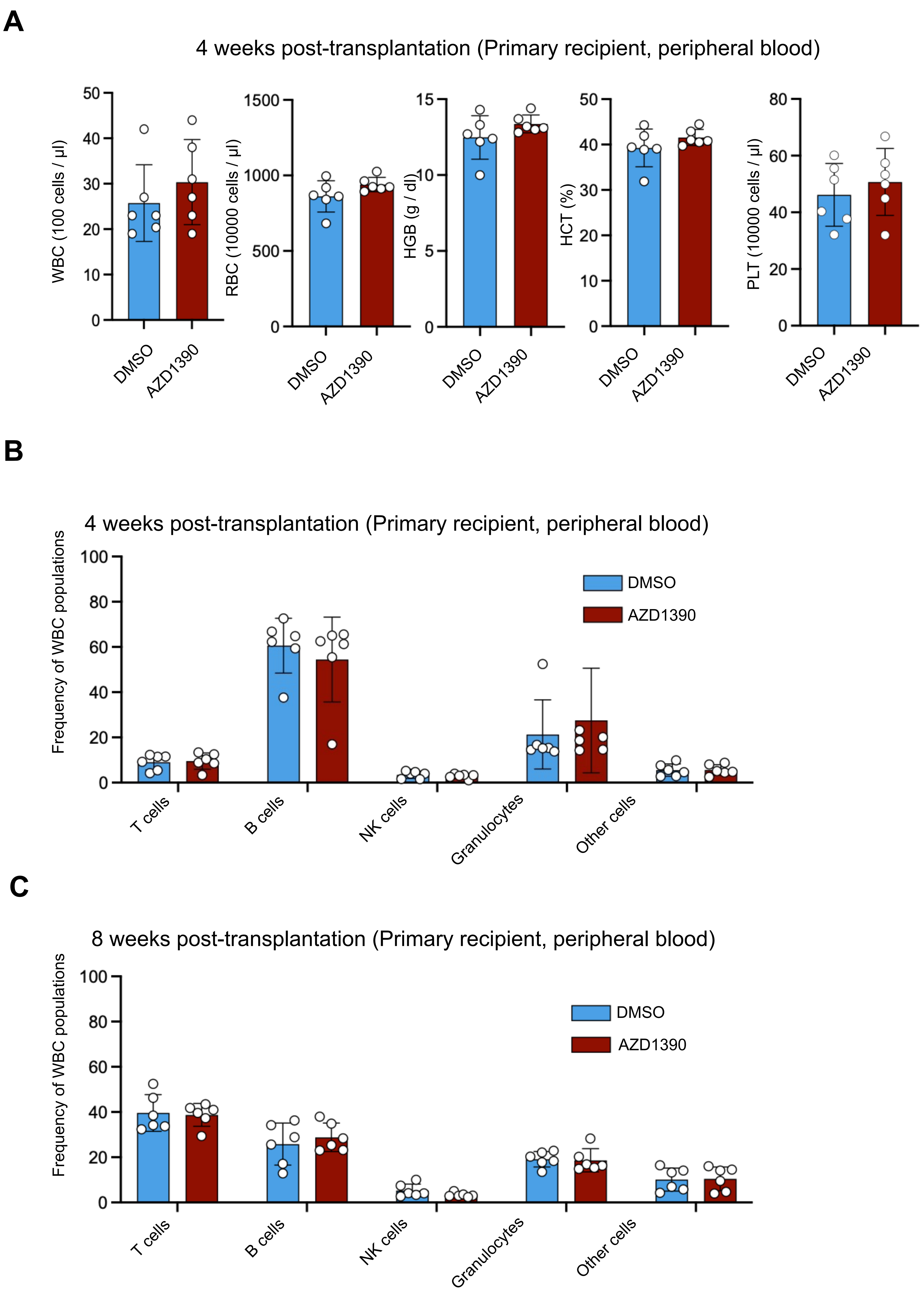

Complete blood count (CBC) and peripheral blood white blood cell (PB WBC) compartment in recipient mice. (A) CBC results 4 weeks post-transplantation. (B) PB WBC compartment 4 weeks post-transplantation and (C) 8 weeks post-transplantation.

Figure S5.

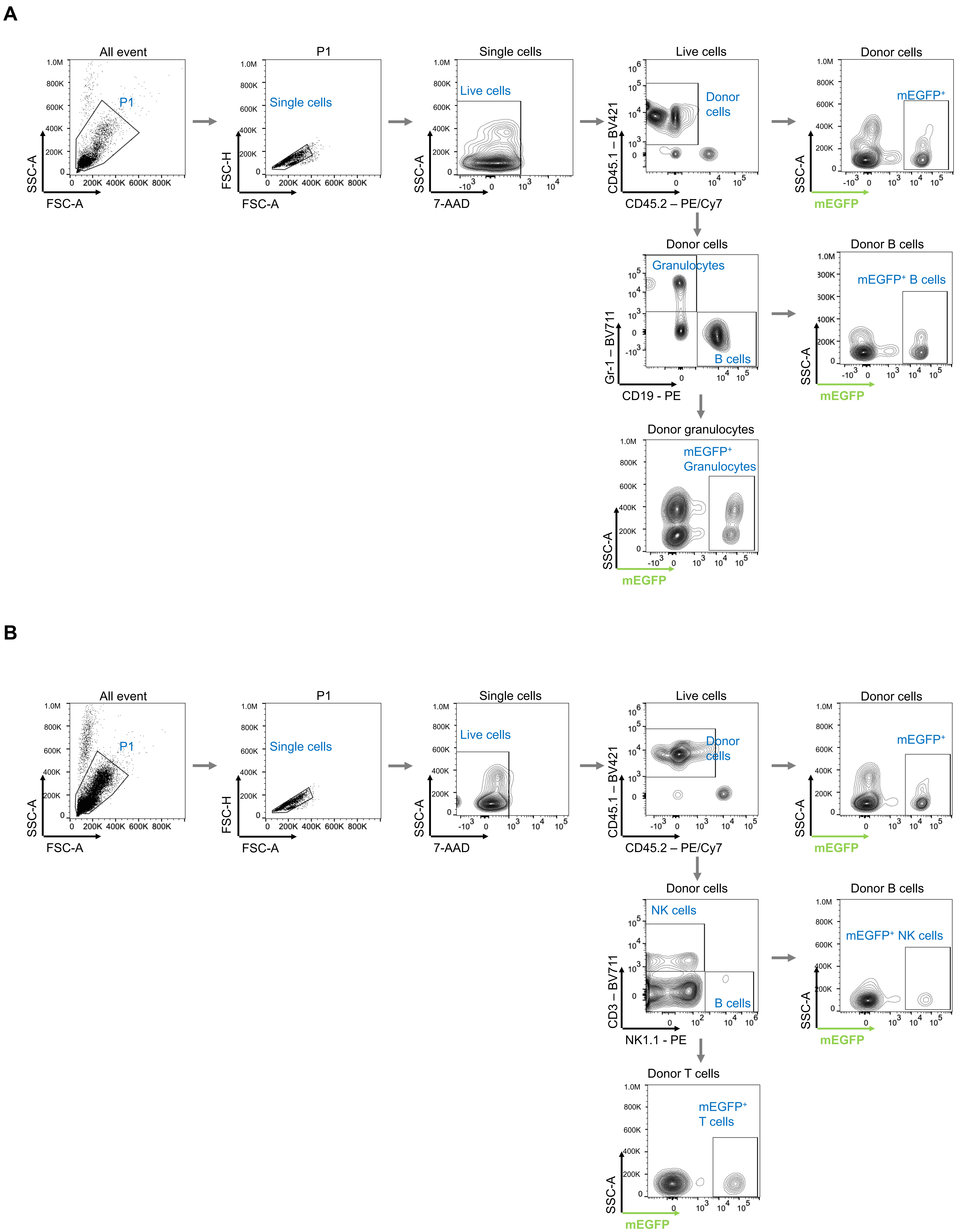

Gating strategy for flow cytometry analysis to evaluate KI efficiency in peripheral blood white blood cells.

(A) Granulocytes and B cells, (B) T and NK cells.

Figure S6.

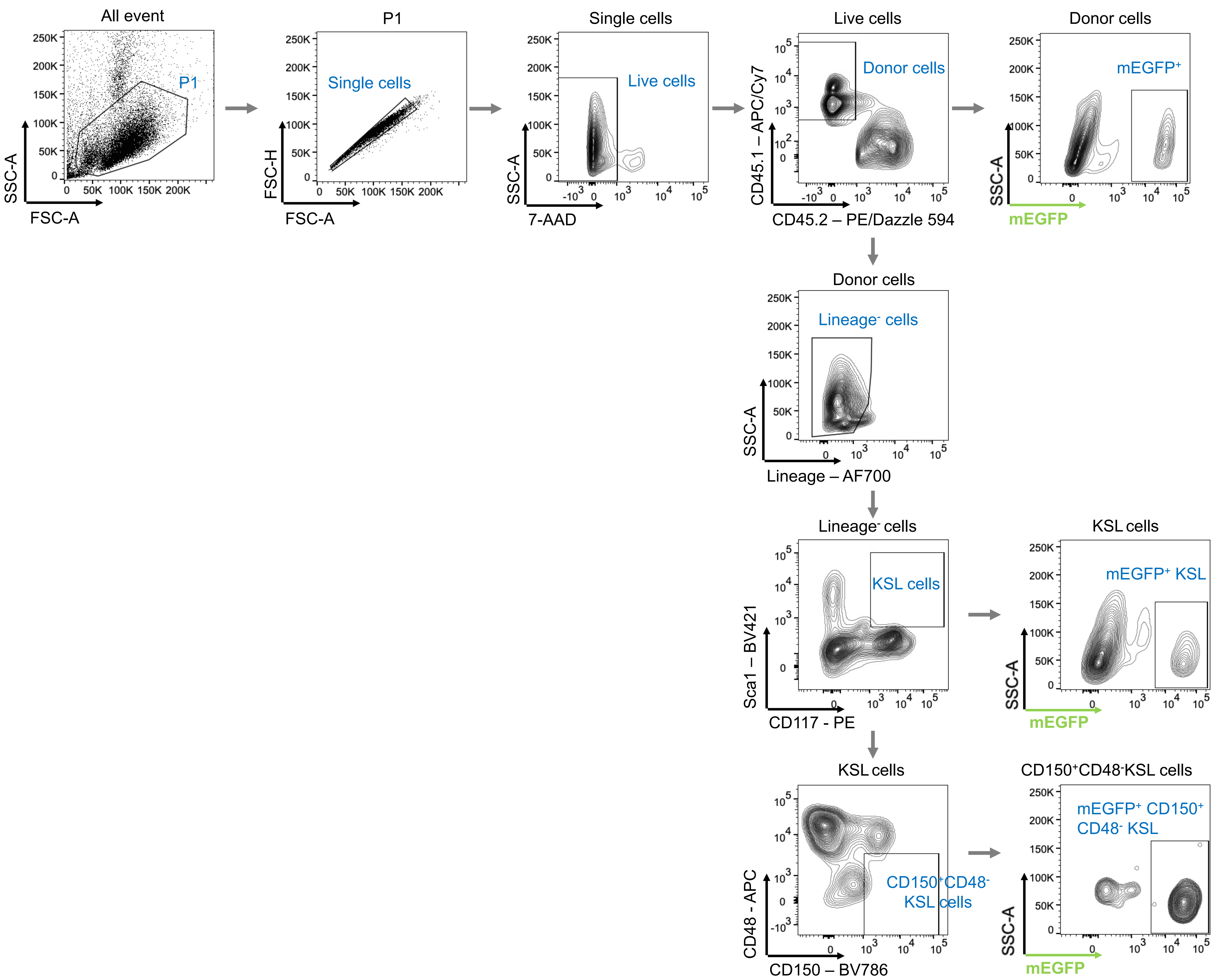

Gating strategy for flow cytometry analysis to evaluate KI efficiency in bone marrow cells.

Figure S7.

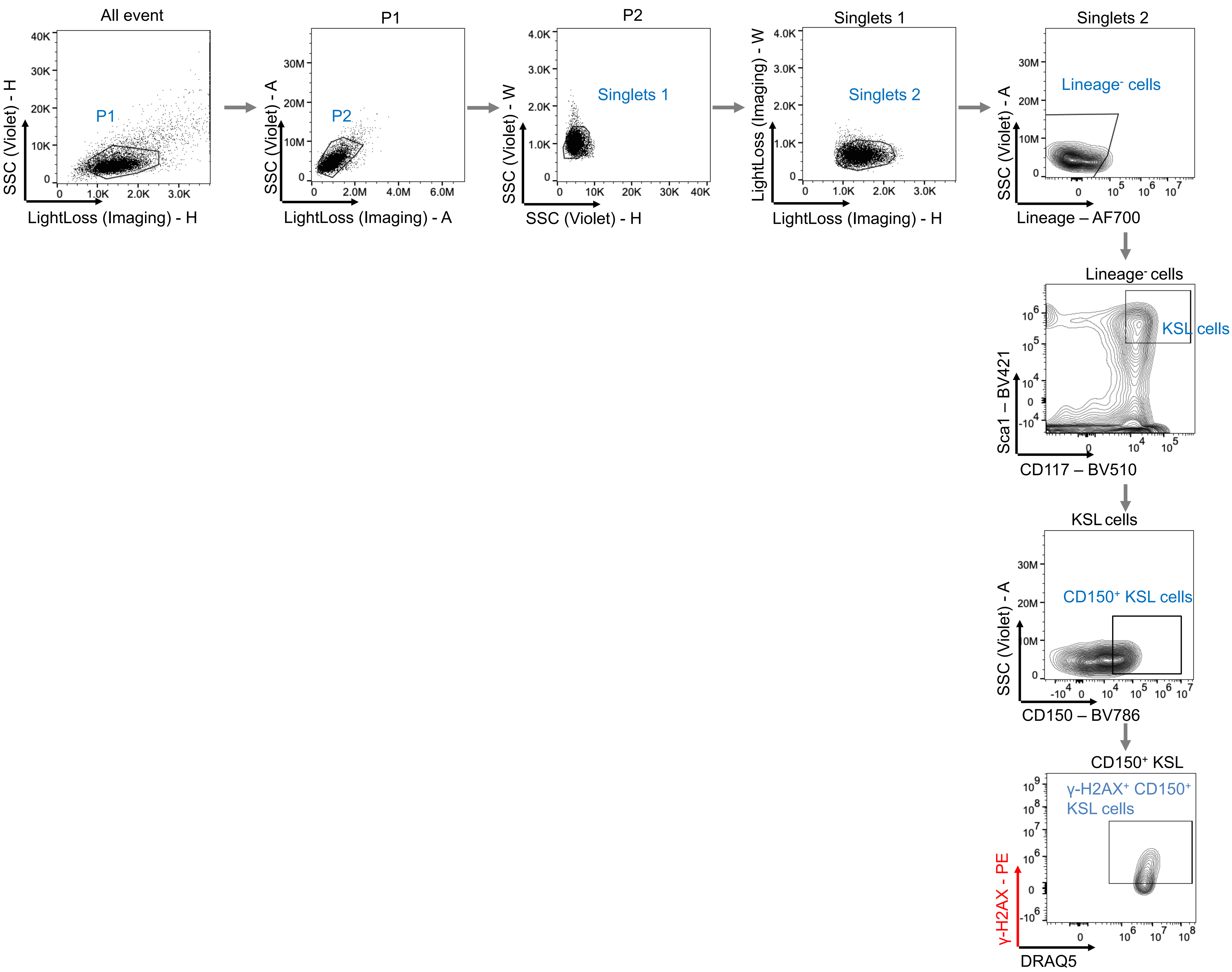

Gating strategy for flow cytometry analysis to evaluate intracellular γ-H2AX level.

Figure S8.

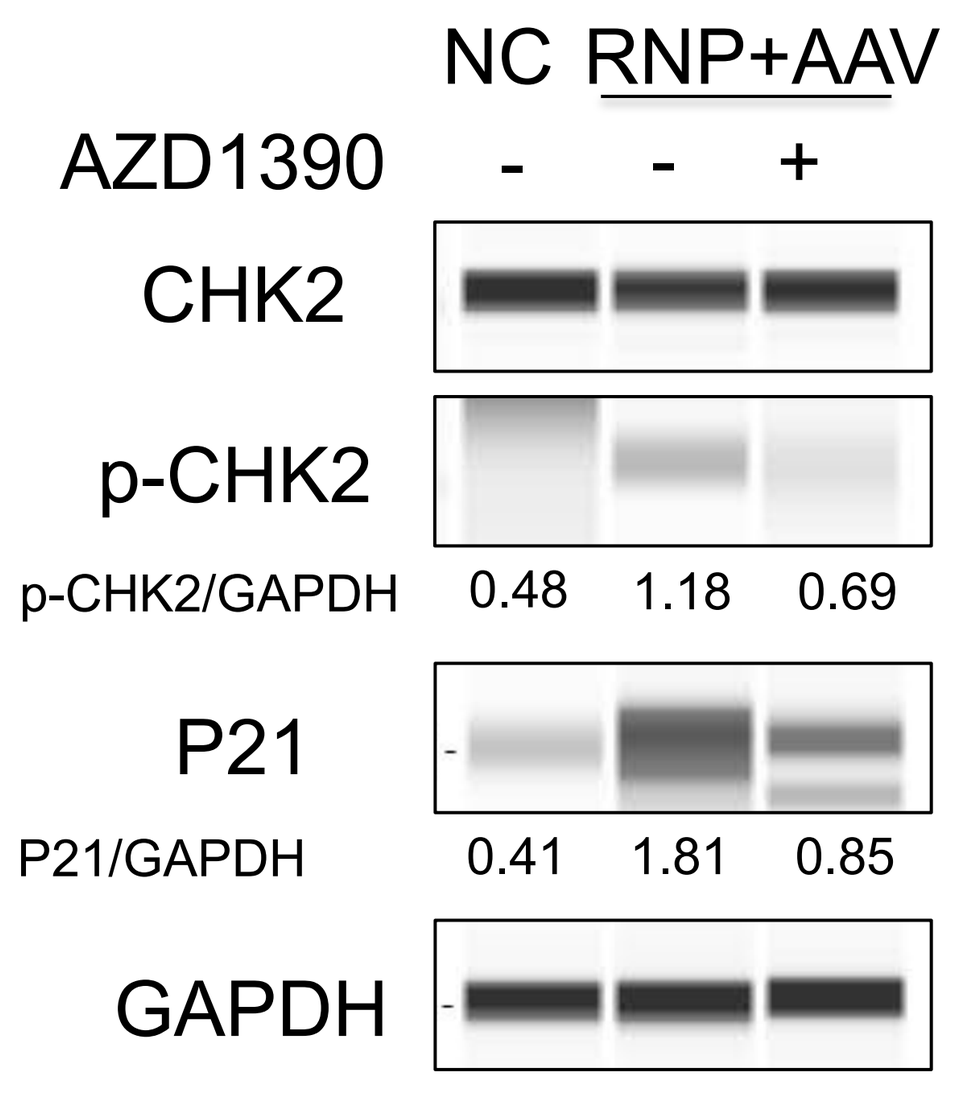

ATM inhibition suppresses CHK2 and following p21 activation. Western blotting results showing genome editing with Cas9/RNP and AAV donor DNA activates CHK2 (Thr-68) and subsequent P21 at 4 hours post-genome editing. 10 nM AZD1390 treatment suppresses the activation of CHK2 and P21.

Figure S9.

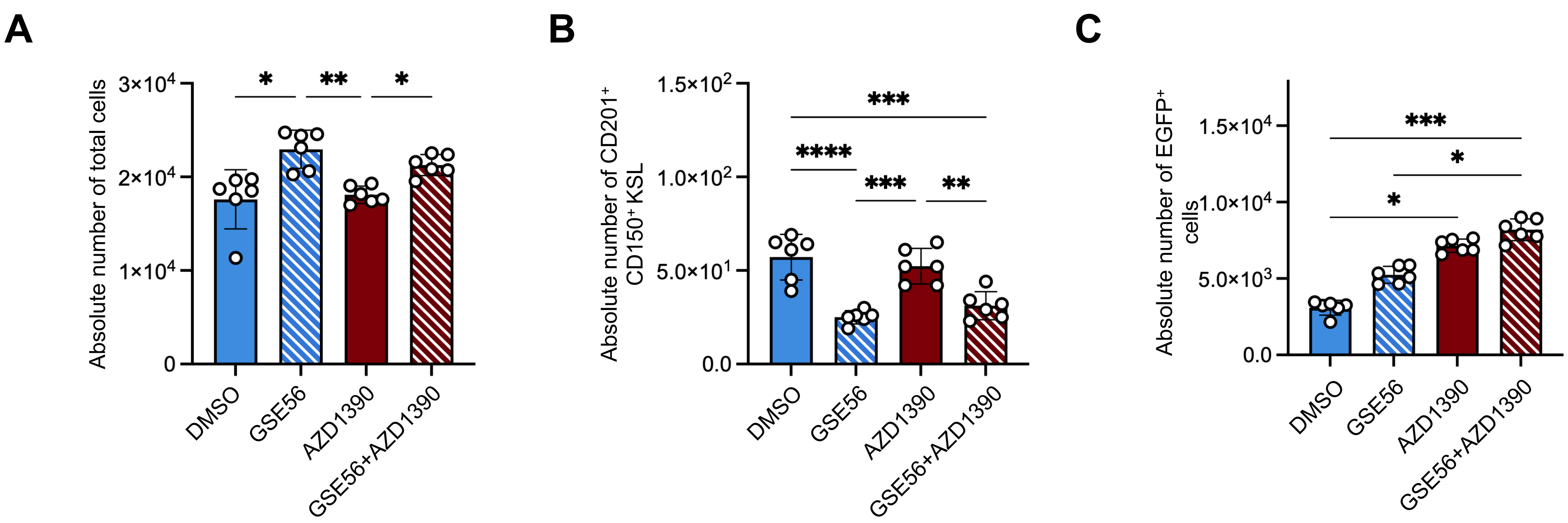

Absolute number of genome-edited cells after p53 inhibition and ATM inhibition. (A) Absolute number of total cells 3-day post-nucleofection. Data are presented as the mean ± SD (n=6). Dunn's multiple comparisons tests were conducted. DMSO vs GSE56, P = 0.0173, DMSO vs AZD1390, P > 0.9999, DMSO vs GSE56+AZD1390, P = 0.1485, GSE56 vs AZD1390, P = 0.0331, GSE56 vs GSE56+AZD1390, P > 0.9999, AZD1390 vs GSE56+AZD1390, P = 0.0374. (B) Absolute number of $CD150^+$ $CD201^+$ KSL cells 3-day post-nucleofection. Data are presented as the mean ± SD (n=6). Tukey's multiple comparisons tests were conducted. DMSO vs GSE56, P < 0.0001, DMSO vs AZD1390, P = 0.7759, DMSO vs GSE56+AZD1390, P = 0.0003, GSE56 vs AZD1390, P = 0.0002, GSE56 vs GSE56+AZD1390, P =0.6228, AZD1390 vs GSE56+AZD1390, P = 0.0024. (C) Absolute number of $mEGFP^+$ cells, Dunn's multiple comparisons test was conducted. Data are presented as the mean ± SD (n=6). DMSO vs GSE56, P = 0.08499; DMSO vs AZD1390, P = 0.0132; DMSO vs GSE56+AZD1390, P = 0.0001; GSE56 vs AZD1390, P = 0.6681; GSE56 vs GSE56+AZD1390, P = 0.0291; AZD1390 vs GSE56+AZD1390, P > 0.9999.

Figure S10.

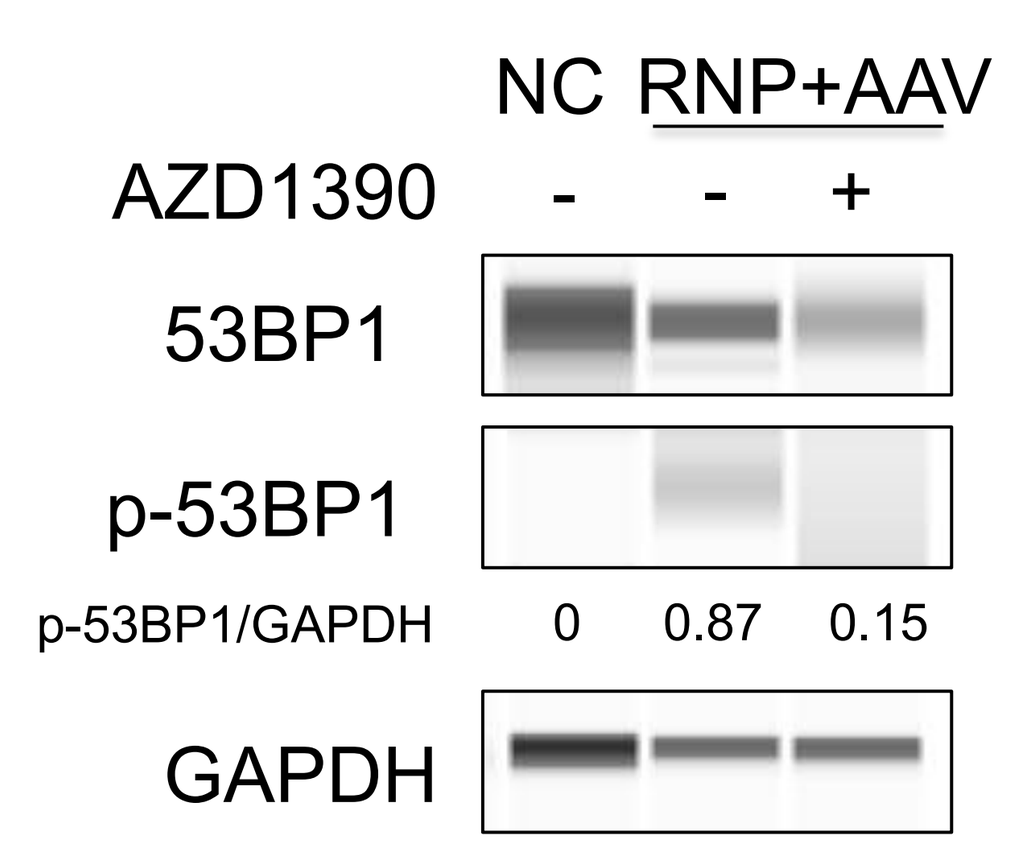

ATM inhibition suppresses 53BP1 activation. Western blotting results showing genome editing with Cas9/RNP and AAV donor DNA activates of 53BP1 at 4 hours post-genome editing. 10 nM AZD1390 treatment suppresses the activation of 53BP1.

Figure S11.

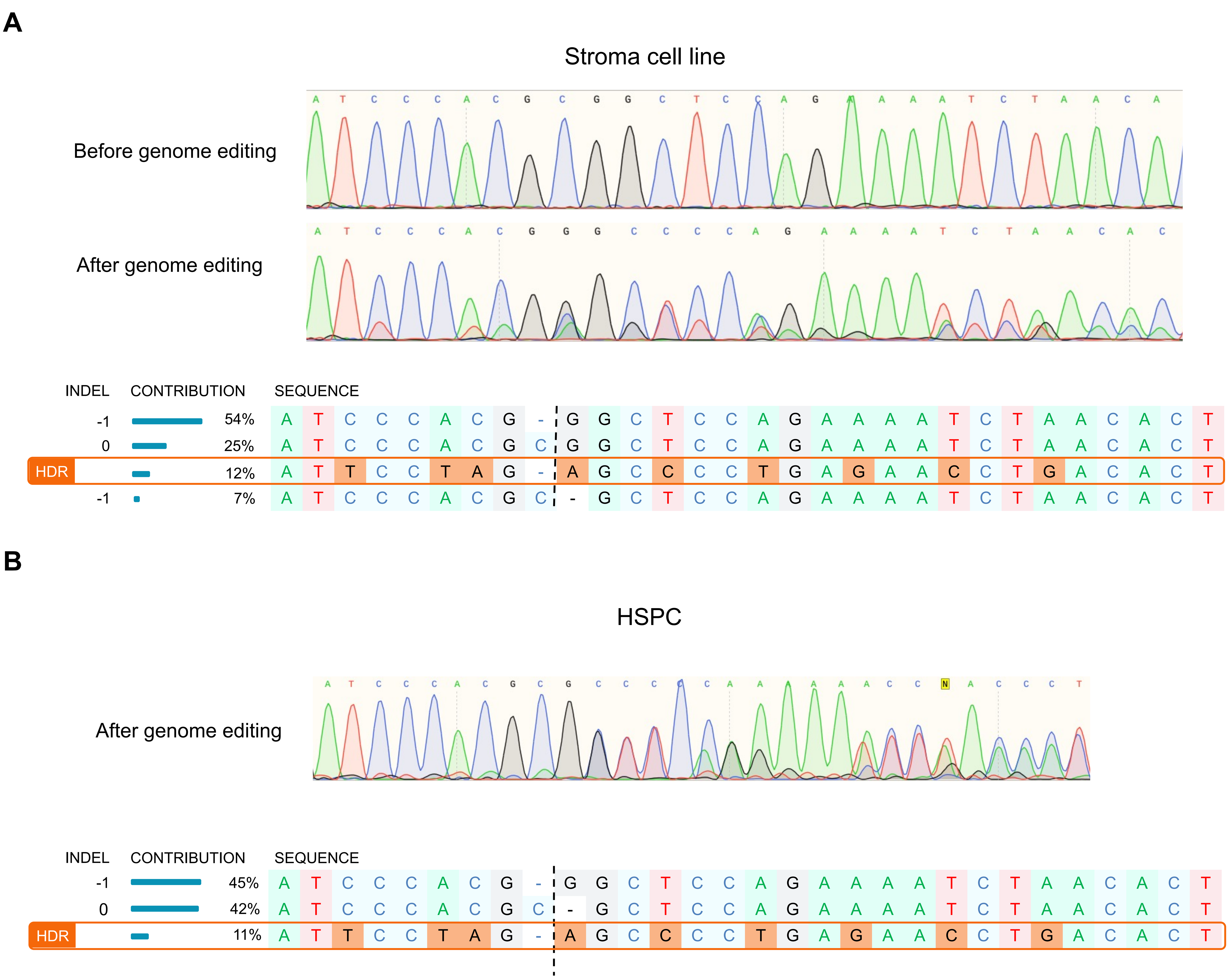

Representative for sequencing results and analysis of HDR by sequencing trace file for *Il2rg* correction. (A) BM-derived stroma cells, (B) HSPCs.

Figure S12.

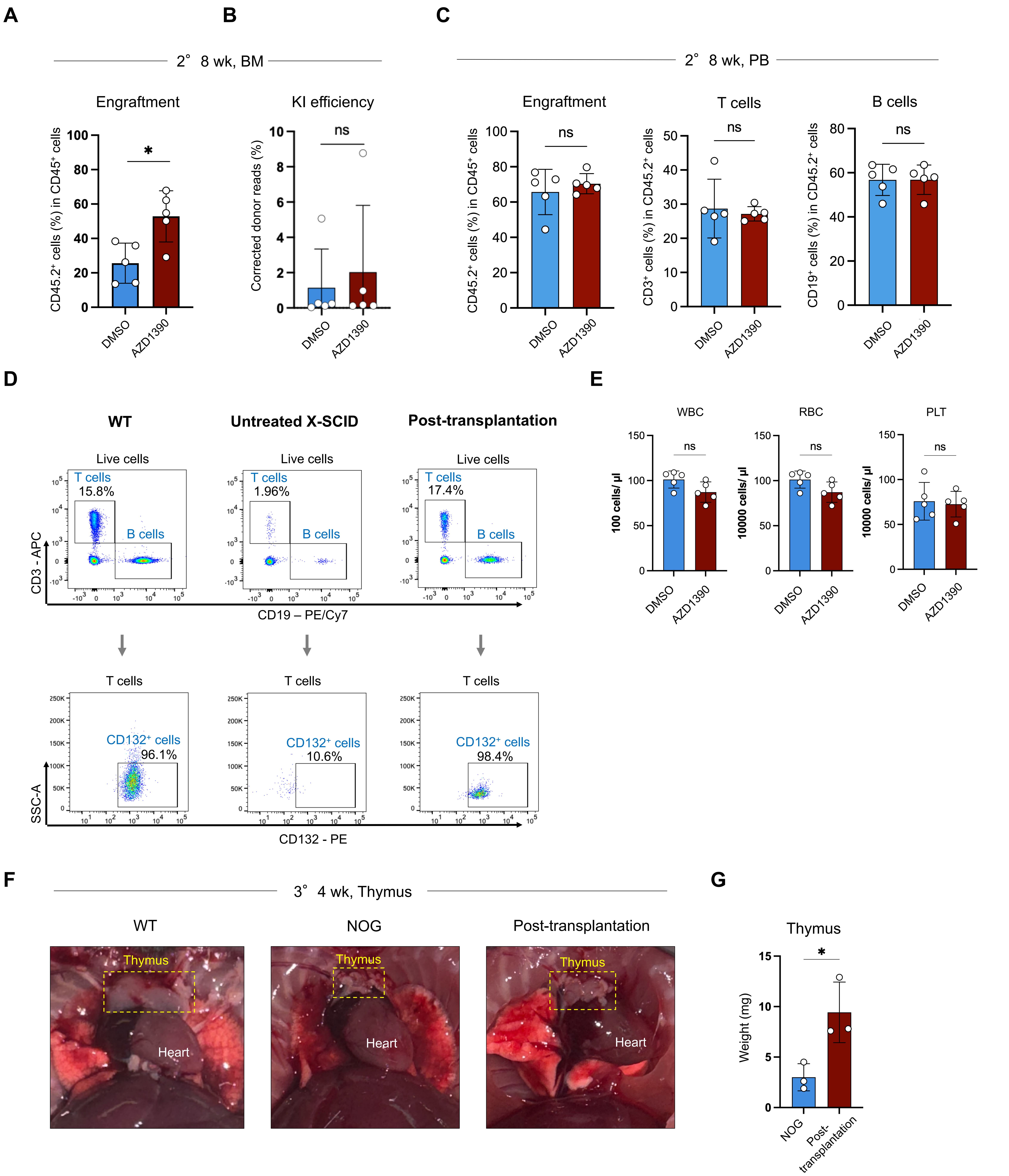

Serial transplantation demonstrates hematopoietic and lymphoid reconstitution following correction of the *Il2rg* mutation. (A) Donor-derived bone marrow (BM) engraftment in secondary recipients at 8 weeks post-transplantation, determined by flow cytometry. (B) KI efficiency in donor-derived BM cells of secondary recipients, determined by amplicon deep sequencing. Corrected donor-derived reads were quantified by analysis of the *Il2rg* target locus. (C) Quantification of donor-derived T- and B-cell frequencies in peripheral blood at 8 weeks post-transplantation. (D) Representative flow cytometry plots showing T- and B-cell reconstitution in the peripheral blood of secondary recipients. (E) Complete blood count analysis of secondary recipients demonstrating restoration of normal hematopoiesis following transplantation of genome-edited HSCs. (F) Representative images of thymic reconstitution in tertiary recipients at 4 weeks post-transplantation, demonstrating sustained lymphoid reconstitution following correction of the *Il2rg* mutation. (G) Weight of thymus from tertiary recipient at 4 weeks post-transplantation. Data are shown as the mean ± standard deviation (n=3). Unpaired t test was conducted. P = 0.0483.

Figure S13.

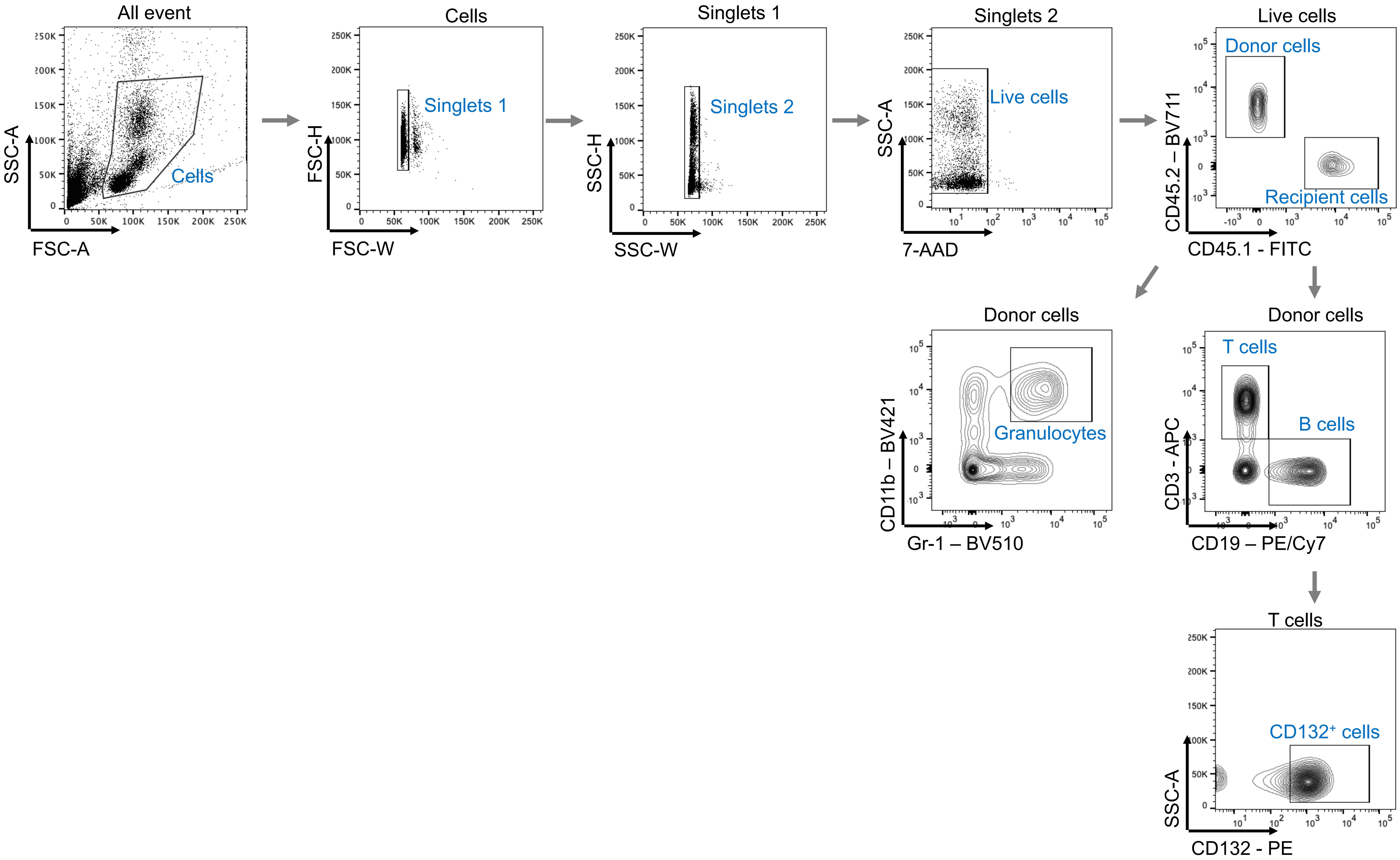

Gating strategy for flow cytometry analysis to evaluate the phenotypical correction of *Il2rg* KO cells

Figure S14.

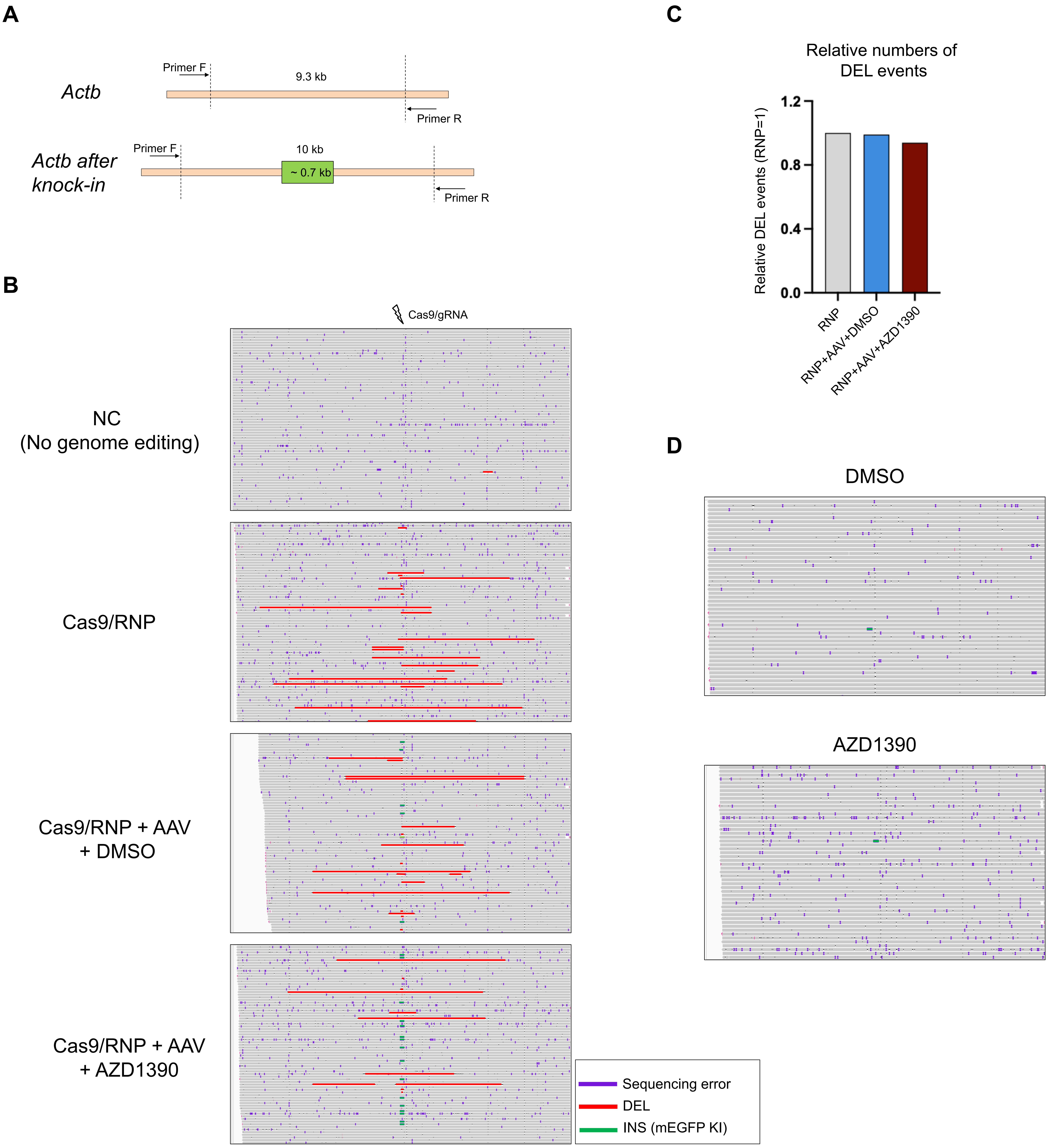

Long-read nanopore sequencing results. (A) Schematic of the workflow for long-read nanopore sequencing. (B) IGV snapshot of representative reads from NC, RNP, RNP+AAV and RNP+AAV+AZD1390 groups following *ex vivo* genome editing. Red line represents deletion events (DEL), green line represents insertion events (mEGFP KI). (C) Relative number of DEL events detected by Dajin2; DEL>41 bp, RNP = 1. (D) IGV snapshots of representative reads from the DMSO and AZD1390 groups using bone marrow DNA from secondary recipients.

Figure S15.

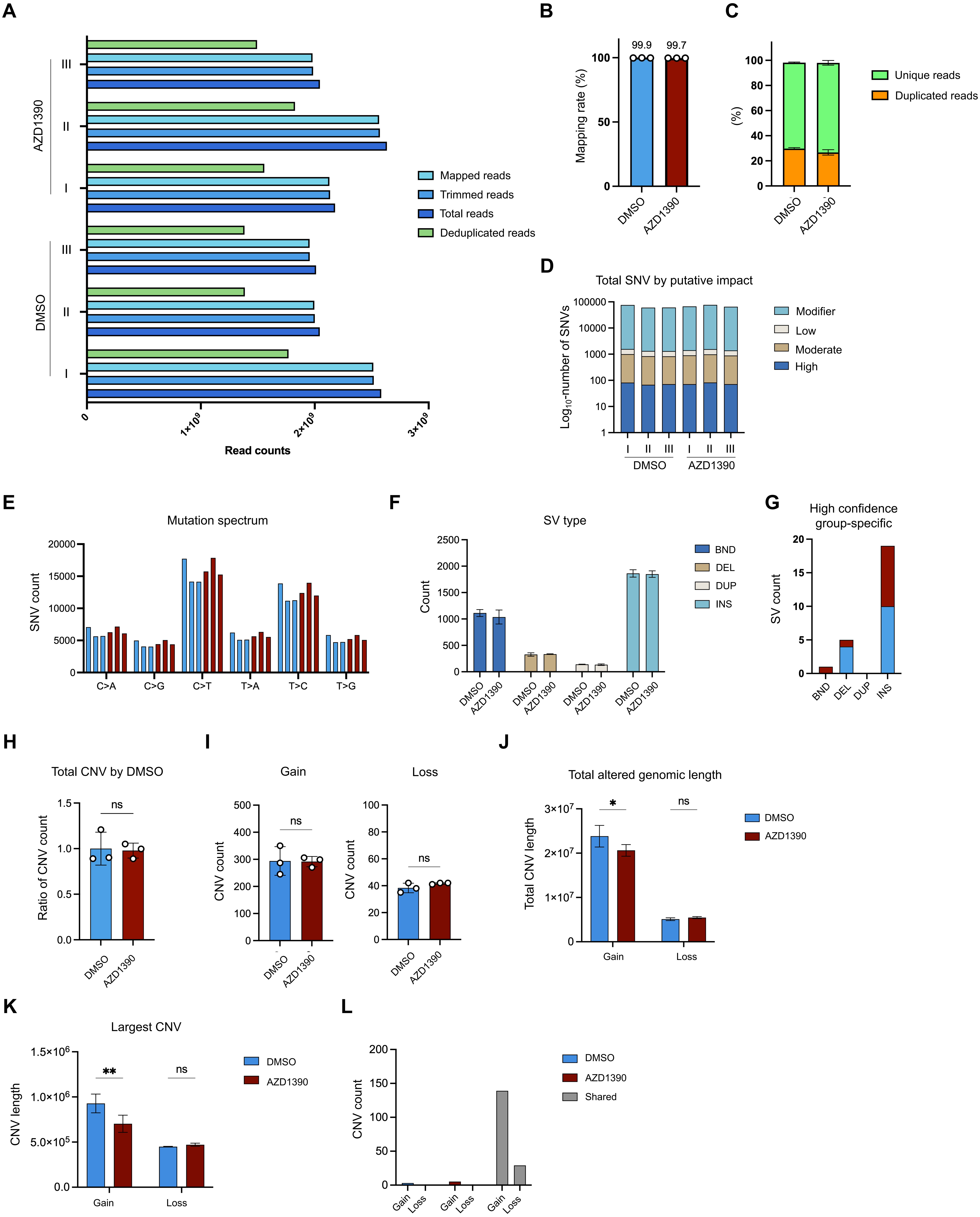

Whole genome sequencing results. (A) Read counts of samples . (B) Mapping rates (C) Read counts of duplicated and unique reads. Duplicated reads are excluded from the analysis. (D) Total single nucleotide variant (SNV) counts by putative impact. (E) SNV mutation spectrum. (F) Structural variant (SV) types (G) High confidence SV types. (H) Ratio of high confidence CNVs. (I) CNV gain and loss count by sample (J) Total altered genomic length (K) Largest CNV size (L) recurrent CNV summary; DMSO-specific, AZD1390-specific and shared.

Figure S16.

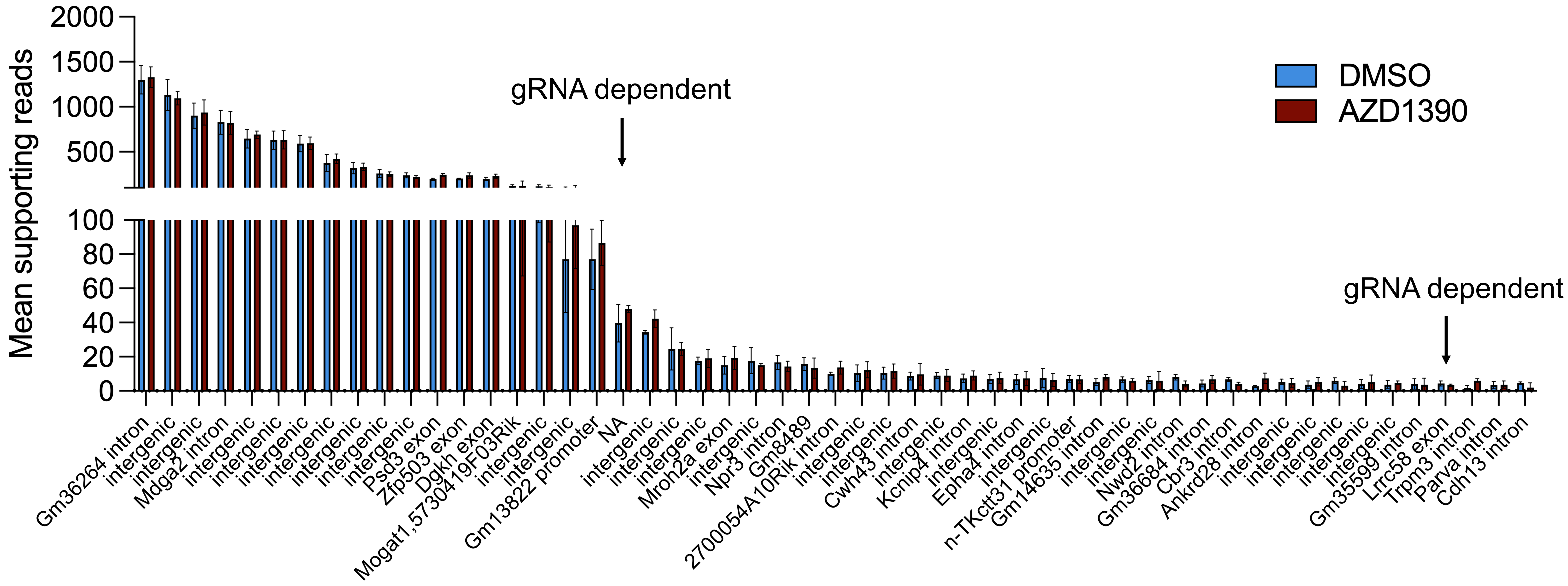

High confidence AAV integrated sites. Data are shown as the mean ± SD (n=3). Šídák's multiple comparisons test was conducted. There were no statistically significant comparison were found between DMSO vs AZD1390 groups. Two gRNA dependent AAV integration events and 51 gRNA-independent AAV integration events were detected.

Figure S17.

A

	Plasmid	AAV
Total barcode	2.4×10^6	5.5×10^5
Shannon diversity index	11.89	11.42
Simpson diversity index	0.99	0.99

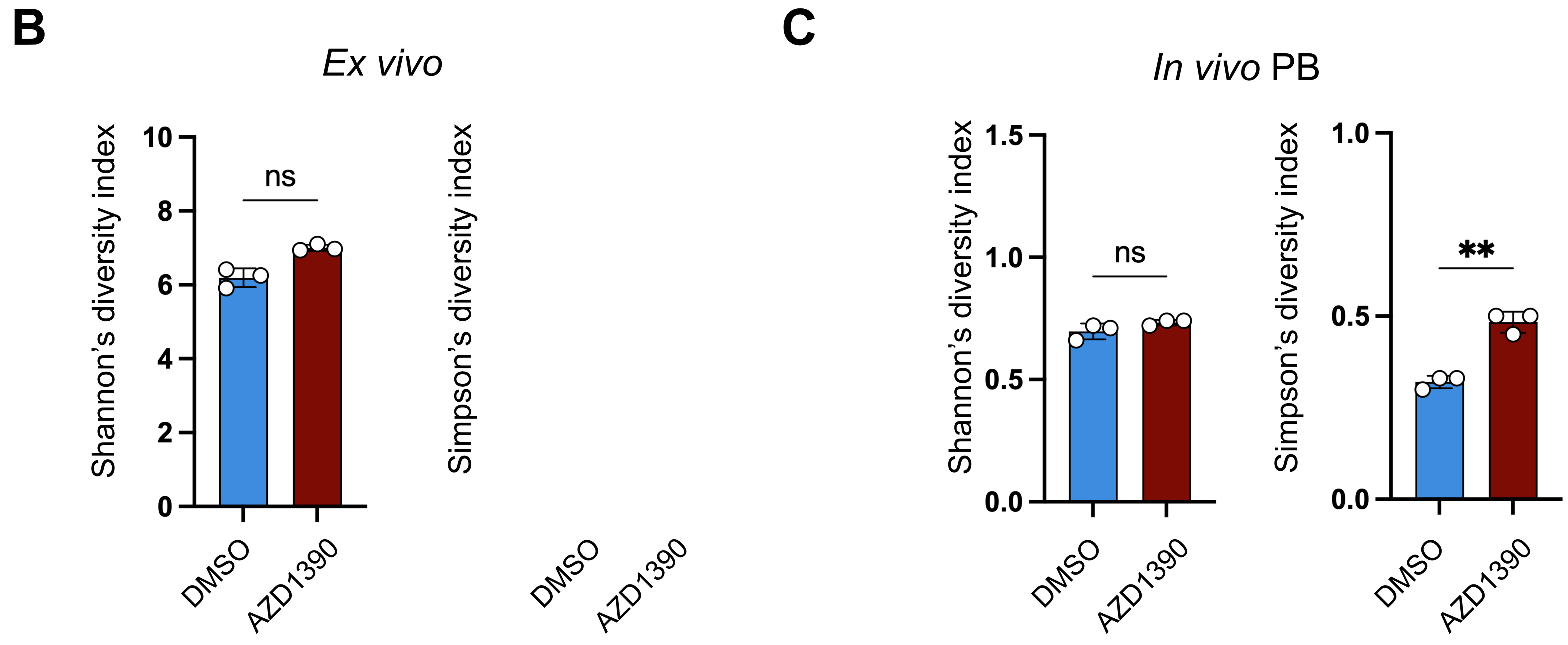

Barcoded clonal tracking results. (A) Information about barcode library in plasmid and AAV donor DNA. (B) Shannon's and Simpson's diversity indices in *ex vivo* genome-edited cells. For Shannon's diversity index, Mann-Whitney t-test was conducted. Data are shown as the mean ± standard deviation (SD) (n=3). P = 0.1; For Simpson's diversity index, Mann-Whitney t-test was conducted. Data are shown as the mean ± SD (n=3). P > 0.9999 (C) Shannon's and Simpson's diversity indices in peripheral blood of recipient mice. For Shannon's diversity index, Mann-Whitney t-test was conducted. Data are shown as the mean ± SD (n=3). P = 0.2; For Simpson's diversity index, unpaired t-test was conducted. Data are shown as the mean ± SD (n=3). P = 0.0025.

Table S1. Read-level reinspection of the 88 group-specific structural variant (SV) calls identified by the original Manta clustering analysis. The table lists the 57 Group 1 and 31 Group 2 calls together with SV class, genomic position, original read support, and the result of secondary BAM reinspection. For BNDs, exact junction evidence linking the expected breakpoint pair was evaluated across all six BAM files. For DEL and DUP calls, exact breakpoint-associated read evidence was compared between groups. For INS calls, Manta-derived insertion-sequence fragments were searched in breakpoint-proximal BAM reads because Manta insertions may be reconstructed by local assembly and are not necessarily represented by a single CIGAR insertion. Detection of evidence in both groups was interpreted as failure to support the original group-specific designation. Absence of exact evidence was considered unresolved and compatible with a mapping/calling artifact, rather than proof of a false-positive call. None of the 88 SVs was independently validated by an orthogonal experimental method; therefore, BAM-supported calls should not be interpreted as experimentally confirmed true positives.

ID	Group	Chromosome	Position	SV type	SV length	Support	Final classification	Explanation
1	DMSO	chr1	55867035	BND	NA	40	Likely artifact / unresolved	No exact ±20-bp junction-supporting reads were detected in either group. This is compatible with a mapping/calling or threshold-related artifact, but no orthogonal validation was performed.
2	DMSO	chr1	1.43E+08	BND	NA	31	Not group-specific	Exact junction-supporting reads were detected in both groups; therefore the original group-specific designation is not supported.
3	DMSO	chr1	1.86E+08	BND	NA	23	Not group-specific	Exact junction-supporting reads were detected in both groups; therefore the original group-specific designation is not supported.
4	DMSO	chr2	4224759	BND	NA	34	Likely artifact / unresolved	No exact ±20-bp junction-supporting reads were detected in either group. This is compatible with a mapping/calling or threshold-related artifact, but no orthogonal validation was performed.
5	DMSO	chr2	1.12E+08	BND	NA	25	Not group-specific	Exact junction-supporting reads were detected in both groups; therefore the original group-specific designation is not supported.
6	DMSO	chr2	1.56E+08	BND	NA	29	Not group-specific	Exact junction-supporting reads were detected only in the opposite group, not in the originally assigned group.
7	DMSO	chr2	1.56E+08	INS	218	65	Group specificity not confirmed	Manta insertion-sequence fragments were detected in BAM reads from both groups. Sequence matching therefore does not independently confirm the original group-specific designation; it also does not prove that the complete insertion allele is present in both groups.
8	DMSO	chr2	1.62E+08	BND	NA	22	Not group-specific	Exact junction-supporting reads were detected in both groups; therefore the original group-specific designation is not supported.
9	DMSO	chr3	13383225	INS	95	24	Group specificity not confirmed	Manta insertion-sequence fragments were detected in BAM reads from both groups. Sequence matching therefore does not independently confirm the original group-specific designation; it also does not prove that the complete insertion allele is present in both groups.
10	DMSO	chr3	15832812	BND	NA	46	Likely artifact / unresolved	No exact ±20-bp junction-supporting reads were detected in either group. This is compatible with a mapping/calling or threshold-related artifact, but no orthogonal validation was performed.
11	DMSO	chr3	76493607	BND	NA	34	BAM-supported candidate	Exact junction-supporting reads were detected only in the originally assigned group. This supports group specificity bioinformatically, but the event was not independently confirmed by an orthogonal assay.
12	DMSO	chr4	4182498	INS	231	34	Group specificity not confirmed	Manta insertion-sequence fragments were detected in BAM reads from both groups. Sequence matching therefore does not independently confirm the original group-specific designation; it also does not prove that the complete insertion allele is present in both groups.
13	DMSO	chr4	26783861	INS	NA	202	Unresolved / possible artifact	No insertion-sequence support was detected in either group with the sequence-level BAM test. This raises concern for an artifact but does not experimentally disprove the Manta call.
14	DMSO	chr4	47210393	INS	146	81	Group specificity not confirmed	Manta insertion-sequence fragments were detected in BAM reads from both groups. Sequence matching therefore does not independently confirm the original group-specific designation; it also does not prove that the complete insertion allele is present in both groups.
15	DMSO	chr4	50184195	BND	NA	25	Not group-specific	Exact junction-supporting reads were detected in both groups; therefore the original group-specific designation is not supported.
16	DMSO	chr4	89452925	INS	119	114	Group specificity not confirmed	Manta insertion-sequence fragments were detected in BAM reads from both groups. Sequence matching therefore does not independently confirm the original group-specific designation; it also does not prove that the complete insertion allele is present in both groups.
17	DMSO	chr5	45264263	INS	NA	41	Group specificity not confirmed	Manta insertion-sequence fragments were detected in BAM reads from both groups. Sequence matching therefore does not independently confirm the original group-specific designation; it also does not prove that the complete insertion allele is present in both groups.
18	DMSO	chr5	48896887	BND	NA	41	Likely artifact / unresolved	No exact ±20-bp junction-supporting reads were detected in either group. This is compatible with a mapping/calling or threshold-related artifact, but no orthogonal validation was performed.
19	DMSO	chr5	76574911	DEL	-64	62	Unresolved	No exact DEL-associated evidence was detected in either group using this read-level test. This does not experimentally disprove the Manta call.
20	DMSO	chr5	77555066	INS	147	32	Group specificity not confirmed	Manta insertion-sequence fragments were detected in BAM reads from both groups. Sequence matching therefore does not independently confirm the original group-specific designation; it also does not prove that the complete insertion allele is present in both groups.
21	DMSO	chr5	92654903	INS	145	30	Group specificity not confirmed	Manta insertion-sequence fragments were detected in BAM reads from both groups. Sequence matching therefore does not independently confirm the original group-specific designation; it also does not prove that the complete insertion allele is present in both groups.

22	DMSO	chr5	1.13E+08	INS	NA	130	Group specificity not confirmed	Manta insertion-sequence fragments were detected in BAM reads from both groups. Sequence matching therefore does not independently confirm the original group-specific designation; it also does not prove that the complete insertion allele is present in both groups.
23	DMSO	chr5	1.17E+08	BND	NA	25	Not group-specific	Exact junction-supporting reads were detected in both groups; therefore the original group-specific designation is not supported.
24	DMSO	chr5	1.17E+08	BND	NA	28	Not group-specific	Exact junction-supporting reads were detected in both groups; therefore the original group-specific designation is not supported.
25	DMSO	chr5	1.35E+08	BND	NA	47	BAM-supported candidate	Exact junction-supporting reads were detected only in the originally assigned group. This supports group specificity bioinformatically, but the event was not independently confirmed by an orthogonal assay.
26	DMSO	chr6	26067421	INS	84	55	Group specificity not confirmed	Manta insertion-sequence fragments were detected in BAM reads from both groups. Sequence matching therefore does not independently confirm the original group-specific designation; it also does not prove that the complete insertion allele is present in both groups.
27	DMSO	chr6	42378713	BND	NA	29	Not group-specific	Exact junction-supporting reads were detected only in the opposite group, not in the originally assigned group.
28	DMSO	chr7	73843120	BND	NA	34	BAM-supported candidate	Exact junction-supporting reads were detected only in the originally assigned group. This supports group specificity bioinformatically, but the event was not independently confirmed by an orthogonal assay.
29	DMSO	chr7	1.31E+08	INS	89	25	Group specificity not confirmed	Manta insertion-sequence fragments were detected in BAM reads from both groups. Sequence matching therefore does not independently confirm the original group-specific designation; it also does not prove that the complete insertion allele is present in both groups.
30	DMSO	chr9	41113535	DUP	55	12	Not group-specific	Exact DUP-associated BAM evidence was detected in both groups, so the original group-specific designation is not supported.
31	DMSO	chr9	58148339	BND	NA	19	Not group-specific	Exact junction-supporting reads were detected in both groups; therefore the original group-specific designation is not supported.
32	DMSO	chr9	1.09E+08	DEL	-226	51	Unresolved	No exact DEL-associated evidence was detected in either group using this read-level test. This does not experimentally disprove the Manta call.
33	DMSO	chr10	84491191	DEL	-56	53	Unresolved	No exact DEL-associated evidence was detected in either group using this read-level test. This does not experimentally disprove the Manta call.
34	DMSO	chr10	92986638	INS	NA	39	Group specificity not confirmed	Manta insertion-sequence fragments were detected in BAM reads from both groups. Sequence matching therefore does not independently confirm the original group-specific designation; it also does not prove that the complete insertion allele is present in both groups.
35	DMSO	chr11	48088590	INS	329	74	Group specificity not confirmed	Manta insertion-sequence fragments were detected in BAM reads from both groups. Sequence matching therefore does not independently confirm the original group-specific designation; it also does not prove that the complete insertion allele is present in both groups.
36	DMSO	chr11	66732272	INS	121	18	Group specificity not confirmed	Manta insertion-sequence fragments were detected in BAM reads from both groups. Sequence matching therefore does not independently confirm the original group-specific designation; it also does not prove that the complete insertion allele is present in both groups.
37	DMSO	chr12	38043960	INS	NA	54	Group specificity not confirmed	Manta insertion-sequence fragments were detected in BAM reads from both groups. Sequence matching therefore does not independently confirm the original group-specific designation; it also does not prove that the complete insertion allele is present in both groups.
38	DMSO	chr12	59780262	INS	NA	65	Group specificity not confirmed	Manta insertion-sequence fragments were detected in BAM reads from both groups. Sequence matching therefore does not independently confirm the original group-specific designation; it also does not prove that the complete insertion allele is present in both groups.
39	DMSO	chr13	78422725	BND	NA	20	Not group-specific	Exact junction-supporting reads were detected in both groups; therefore the original group-specific designation is not supported.
40	DMSO	chr13	81617795	BND	NA	34	Not group-specific	Exact junction-supporting reads were detected in both groups; therefore the original group-specific designation is not supported.
41	DMSO	chr13	1.08E+08	BND	NA	23	Not group-specific	Exact junction-supporting reads were detected in both groups; therefore the original group-specific designation is not supported.
42	DMSO	chr14	23435680	BND	NA	41	Likely artifact / unresolved	No exact ±20-bp junction-supporting reads were detected in either group. This is compatible with a mapping/calling or threshold-related artifact, but no orthogonal validation was performed.
43	DMSO	chr14	27442453	DUP	69	19	Not group-specific	Exact DUP-associated BAM evidence was detected in both groups, so the original group-specific designation is not supported.
44	DMSO	chr14	69034831	INS	NA	54	Group specificity not confirmed	Manta insertion-sequence fragments were detected in BAM reads from both groups. Sequence matching therefore does not independently confirm the original group-specific designation; it also does not prove that the complete insertion allele is present in both groups.
45	DMSO	chr14	74360546	BND	NA	19	Likely artifact / unresolved	No exact ±20-bp junction-supporting reads were detected in either group. This is compatible with a mapping/calling or threshold-related artifact, but no orthogonal validation was performed.
46	DMSO	chr15	49485268	BND	NA	28	Likely artifact / unresolved	No exact ±20-bp junction-supporting reads were detected in either group. This is compatible with a mapping/calling or threshold-related artifact, but no orthogonal validation was performed.

47	DMSO	chr15	53584828	INS	61	31	Group specificity not confirmed	Manta insertion-sequence fragments were detected in BAM reads from both groups. Sequence matching therefore does not independently confirm the original group-specific designation; it also does not prove that the complete insertion allele is present in both groups.
48	DMSO	chr16	24694094	BND	NA	34	Likely artifact / unresolved	No exact ±20-bp junction-supporting reads were detected in either group. This is compatible with a mapping/calling or threshold-related artifact, but no orthogonal validation was performed.
49	DMSO	chr16	32305779	INS	56	15	Group specificity not confirmed	Manta insertion-sequence fragments were detected in BAM reads from both groups. Sequence matching therefore does not independently confirm the original group-specific designation; it also does not prove that the complete insertion allele is present in both groups.
50	DMSO	chr16	78049868	DEL	-55	51	Unresolved	No exact DEL-associated evidence was detected in either group using this read-level test. This does not experimentally disprove the Manta call.
51	DMSO	chr17	25839821	BND	NA	17	Not group-specific	Exact junction-supporting reads were detected in both groups; therefore the original group-specific designation is not supported.
52	DMSO	chr17	81981954	BND	NA	21	Not group-specific	Exact junction-supporting reads were detected in both groups; therefore the original group-specific designation is not supported.
53	DMSO	chr18	75025685	INS	60	43	Group specificity not confirmed	Manta insertion-sequence fragments were detected in BAM reads from both groups. Sequence matching therefore does not independently confirm the original group-specific designation; it also does not prove that the complete insertion allele is present in both groups.
54	DMSO	chr19	14164750	BND	NA	28	Not group-specific	Exact junction-supporting reads were detected in both groups; therefore the original group-specific designation is not supported.
55	DMSO	chrY	3296265	INS	57	26	Group specificity not confirmed	Manta insertion-sequence fragments were detected in BAM reads from both groups. Sequence matching therefore does not independently confirm the original group-specific designation; it also does not prove that the complete insertion allele is present in both groups.
56	DMSO	chr10	59418901	INS	360	78	Group specificity not confirmed	Manta insertion-sequence fragments were detected in BAM reads from both groups. Sequence matching therefore does not independently confirm the original group-specific designation; it also does not prove that the complete insertion allele is present in both groups.
57	DMSO	chr16	84961586	BND	NA	22	Likely artifact / unresolved	No exact ±20-bp junction-supporting reads were detected in either group. This is compatible with a mapping/calling or threshold-related artifact, but no orthogonal validation was performed.
58	AZD1390	chr1	40779466	INS	164	35	Group specificity not confirmed	Manta insertion-sequence fragments were detected in BAM reads from both groups. Sequence matching therefore does not independently confirm the original group-specific designation; it also does not prove that the complete insertion allele is present in both groups.
59	AZD1390	chr1	1.71E+08	DEL	-86	90	Not group-specific	Exact DEL-associated BAM evidence was detected in both groups, so the original group-specific designation is not supported.
60	AZD1390	chr2	1.18E+08	BND	NA	17	Not group-specific	Exact junction-supporting reads were detected in both groups; therefore the original group-specific designation is not supported.
61	AZD1390	chr3	76493593	INS	NA	29	Group specificity not confirmed	Manta insertion-sequence fragments were detected in BAM reads from both groups. Sequence matching therefore does not independently confirm the original group-specific designation; it also does not prove that the complete insertion allele is present in both groups.
62	AZD1390	chr3	1.16E+08	INS	99	37	Group specificity not confirmed	Manta insertion-sequence fragments were detected in BAM reads from both groups. Sequence matching therefore does not independently confirm the original group-specific designation; it also does not prove that the complete insertion allele is present in both groups.
63	AZD1390	chr4	62497873	INS	193	68	Group specificity not confirmed	Manta insertion-sequence fragments were detected in BAM reads from both groups. Sequence matching therefore does not independently confirm the original group-specific designation; it also does not prove that the complete insertion allele is present in both groups.
64	AZD1390	chr5	37642720	INS	112	68	Group specificity not confirmed	Manta insertion-sequence fragments were detected in BAM reads from both groups. Sequence matching therefore does not independently confirm the original group-specific designation; it also does not prove that the complete insertion allele is present in both groups.
65	AZD1390	chr5	53342699	DEL	-82438477	22	Not group-specific	Exact DEL-associated BAM evidence was detected in both groups, so the original group-specific designation is not supported.
66	AZD1390	chr8	63112413	BND	NA	30	Not group-specific	Exact junction-supporting reads were detected in both groups; therefore the original group-specific designation is not supported.
67	AZD1390	chr8	1.27E+08	BND	NA	61	Likely artifact / unresolved	No exact ±20-bp junction-supporting reads were detected in either group. This is compatible with a mapping/calling or threshold-related artifact, but no orthogonal validation was performed.
68	AZD1390	chr9	25355383	BND	NA	26	Not group-specific	Exact junction-supporting reads were detected in both groups; therefore the original group-specific designation is not supported.
69	AZD1390	chr9	57929628	DUP	375	34	Not group-specific	Exact DUP-associated BAM evidence was detected in both groups, so the original group-specific designation is not supported.
70	AZD1390	chr9	62395244	INS	146	74	Group specificity not confirmed	Manta insertion-sequence fragments were detected in BAM reads from both groups. Sequence matching therefore does not independently confirm the original group-specific designation; it also does not prove that the complete insertion allele is present in both groups.
71	AZD1390	chr9	82563272	DEL	-58	20	Not group-specific	Exact DEL-associated BAM evidence was detected in both groups, so the original group-specific designation is not supported.
72	AZD1390	chr10	97572478	INS	59	30	Group specificity not confirmed	Manta insertion-sequence fragments were detected in BAM reads from both groups. Sequence matching therefore does not independently confirm the original group-specific designation; it also does not prove that the complete insertion allele is present in both groups.
73	AZD1390	chr11	29599555	INS	204	55	Group specificity not confirmed	Manta insertion-sequence fragments were detected in BAM reads from both groups. Sequence matching therefore does not independently confirm the original group-specific designation; it also does not prove that the complete insertion allele is present in both groups.

74	AZD1390	chr12	1.08E+08	INS	304	74	Group specificity not confirmed	Manta insertion-sequence fragments were detected in BAM reads from both groups. Sequence matching therefore does not independently confirm the original group-specific designation; it also does not prove that the complete insertion allele is present in both groups.
75	AZD1390	chr13	39462650	BND	NA	33	Likely artifact / unresolved	No exact ±20-bp junction-supporting reads were detected in either group. This is compatible with a mapping/calling or threshold-related artifact, but no orthogonal validation was performed.
76	AZD1390	chr13	1.1E+08	INS	104	79	Group specificity not confirmed	Manta insertion-sequence fragments were detected in BAM reads from both groups. Sequence matching therefore does not independently confirm the original group-specific designation; it also does not prove that the complete insertion allele is present in both groups.
77	AZD1390	chr15	7587087	INS	169	41	Group specificity not confirmed	Manta insertion-sequence fragments were detected in BAM reads from both groups. Sequence matching therefore does not independently confirm the original group-specific designation; it also does not prove that the complete insertion allele is present in both groups.
78	AZD1390	chr15	61815750	INS	105	65	Group specificity not confirmed	Manta insertion-sequence fragments were detected in BAM reads from both groups. Sequence matching therefore does not independently confirm the original group-specific designation; it also does not prove that the complete insertion allele is present in both groups.
79	AZD1390	chr16	3169633	INS	66	26	Group specificity not confirmed	Manta insertion-sequence fragments were detected in BAM reads from both groups. Sequence matching therefore does not independently confirm the original group-specific designation; it also does not prove that the complete insertion allele is present in both groups.
80	AZD1390	chr18	35852436	INS	57	25	Group specificity not confirmed	Manta insertion-sequence fragments were detected in BAM reads from both groups. Sequence matching therefore does not independently confirm the original group-specific designation; it also does not prove that the complete insertion allele is present in both groups.
81	AZD1390	chr18	53636905	DEL	-296	37	Unresolved	No exact DEL-associated evidence was detected in either group using this read-level test. This does not experimentally disprove the Manta call.
82	AZD1390	chr18	64607333	INS	277	30	Group specificity not confirmed	Manta insertion-sequence fragments were detected in BAM reads from both groups. Sequence matching therefore does not independently confirm the original group-specific designation; it also does not prove that the complete insertion allele is present in both groups.
83	AZD1390	chr19	3702737	BND	NA	30	Not group-specific	Exact junction-supporting reads were detected in both groups; therefore the original group-specific designation is not supported.
84	AZD1390	chr19	25349236	DEL	-121	47	Unresolved	No exact DEL-associated evidence was detected in either group using this read-level test. This does not experimentally disprove the Manta call.
85	AZD1390	chr7	96164020	INS	62	35	Group specificity not confirmed	Manta insertion-sequence fragments were detected in BAM reads from both groups. Sequence matching therefore does not independently confirm the original group-specific designation; it also does not prove that the complete insertion allele is present in both groups.
86	AZD1390	chr12	18652410	BND	NA	28	Not group-specific	Exact junction-supporting reads were detected in both groups; therefore the original group-specific designation is not supported.
87	AZD1390	chr19	15796612	INS	193	52	Group specificity not confirmed	Manta insertion-sequence fragments were detected in BAM reads from both groups. Sequence matching therefore does not independently confirm the original group-specific designation; it also does not prove that the complete insertion allele is present in both groups.
88	AZD1390	chrX	1.59E+08	BND	NA	18	Not group-specific	Exact junction-supporting reads were detected in both groups; therefore the original group-specific designation is not supported.

Table S2. List of the ATM inhibitors.

No	Name	Manufacturer	Product code	Concentration for mouse cells
1	KU55933	Selleck	S1092	1 μM
2	KU60019	Selleck	S1570	1 μM
3	AZD1390	Selleck	S8680	10 nM
4	AZ32	Selleck	S8556	1 μM
5	M4076	Selleck	E1057	1 μM

Table S3. List of the antibodies for flow cytometry analysis.

No	Name	Manufacturer	Product code
1	Alexa Fluor® 700 anti-mouse Lineage Cocktail	Biolegend	133313
2	APC anti-mouse CD3 Antibody	Biolegend	100236
3	APC anti-mouse CD48 Antibody	Biolegend	103412
4	APC/Cyanine7 anti-mouse CD45.1 Antibody	Biolegend	110716
6	BD Horizon™ Brilliant Stain Buffer Plus	BD Biosciences	566385
8	BD Horizon™ BV711 Mouse Anti-Mouse CD45.2	BD Biosciences	563685
9	BD Pharmingen™ 7-AAD	BD Biosciences	559925
10	BD Pharmingen™ PE Mouse Anti-H2AX (pS139)	BD Biosciences	562377
12	BD Pharmingen™ PE Mouse IgG1, κ Isotype Control	BD Biosciences	554680
13	BD Pharmingen™ PE Rat Anti-Mouse CD132	BD Biosciences	554457
14	Biotin anti-mouse Lineage Panel	Biolegend	133307
15	Brilliant Violet 421™ anti-mouse CD45.1 Antibody	Biolegend	110731
16	Brilliant Violet 421™ anti-mouse Ly-6A/E (Sca-1) Antibody	Biolegend	108128
17	Brilliant Violet 421™ anti-mouse/human CD11b Antibody	Biolegend	101236
18	Brilliant Violet 421™ Streptavidin	Biolegend	405226
19	Brilliant Violet 510™ anti-mouse CD117 (c-kit) Antibody	Biolegend	135119
20	Brilliant Violet 510™ Rat IgG2b, κ Isotype Ctrl Antibody	Biolegend	400645
21	Brilliant Violet 711™ anti-mouse CD3 Antibody	Biolegend	100241
22	Brilliant Violet 711™ anti-mouse Ly-6G/Ly-6C (Gr-1) Antibody	Biolegend	108443
23	Brilliant Violet 785™ anti-mouse CD150 (SLAM) Antibody	Biolegend	115937
24	CD117 (c-Kit) Monoclonal Antibody (2B8), APC	Invitrogen	17-1171-82
25	CD117 (c-Kit) Monoclonal Antibody (2B8), PE	Invitrogen	12-1171-82
26	Cellstain® DAPI solution	Dojindo	340-07971
27	DRAQ5™	Biolegend	424101
28	FITC anti-mouse CD45.1 Antibody	Biolegend	110706
29	PE anti-mouse CD19 Antibody	Biolegend	115507
30	PE anti-mouse CD201 (EPCR) Antibody	Biolegend	141504
31	PE anti-mouse NK-1.1 Antibody	Biolegend	108707
32	PE/Cyanine7 anti-mouse CD150 (SLAM) Antibody	Biolegend	115914
33	PE/Cyanine7 anti-mouse CD19 Antibody	Biolegend	115520
34	PE/Cyanine7 anti-mouse CD45.2 Antibody	Biolegend	109830
35	PE/Dazzle™ 594 anti-mouse CD45.2 Antibody	Biolegend	109846
36	PerCP/Cyanine5.5 anti-mouse Ly-6A/E (Sca-1) Antibody	Biolegend	108124
37	Zombie NIR™ Fixable Viability Kit	Biolegend	423105

Table S4. List of the antibodies for western blotting analysis. **ç**

No	Name	Manufacturer	Product code
1	Anti-ATM (phospho S1987)	Abcam	ab315019
2	ATM (D2E2) Rabbit mAb	Cell signaling	2873
3	Caspase-3 Antibody	Cell signaling	9662
4	Cleaved Caspase-3 (Asp175) Antibody	Cell signaling	9661
5	GAPDH Antibody (6C5)	Santa Cruz	sc-32233
6	NPM1 Antibody	Cell signaling	3542S
7	p53 (1C12) Mouse mAb	Cell signaling	2524
8	Phospho-Histone H2A.X (Ser139) (20E3) Rabbit mAb	Cell signaling	9718
9	Phospho-NPM1 (Ser4) Polyclonal Antibody	Invitrogen	PA5-106194
10	Phospho-p53 (Ser15) Antibody	Cell signaling	9284
11	Chk2 Antibody	Cell signaling	2662
12	Phospho-Chk2 (Thr68)	Affinity Biosciences	AF3036
13	p21 Waf1/Cip1 Antibody	Cell signaling	64016

References

1. S. Byambaa *et al.*, Generation of novel Il2rg-knockout mice with clustered regularly interspaced short palindromic repeats (CRISPR) and Cas9. *Exp Anim* **69**, 189-198 (2020).
2. F. Nakahara *et al.*, Hes1 immortalizes committed progenitors and plays a role in blast crisis transition in chronic myelogenous leukemia. *Blood* **115**, 2872-2881 (2010).
3. H. Hara, N. Munkh-Erdene, S. Byambaa, Y. Hanazono, Nonviral Ex Vivo Genome Editing in Mouse Bona Fide Hematopoietic Stem Cells with CRISPR/Cas9. *Methods Mol Biol* **2637**, 213-221 (2023).
4. N. Baatartsogt *et al.*, A sensitive and reproducible cell-based assay via secNanoLuc to detect neutralizing antibody against adeno-associated virus vector capsid. *Mol Ther Methods Clin Dev* **22**, 162-171 (2021).
5. Y. Kashiwakura *et al.*, Efficient gene transduction in pigs and macaques with the engineered AAV vector AAV.GT5 for hemophilia B gene therapy. *Mol Ther Methods Clin Dev* **30**, 502-514 (2023).
6. G. Schiroli *et al.*, Precise Gene Editing Preserves Hematopoietic Stem Cell Function following Transient p53-Mediated DNA Damage Response. *Cell Stem Cell* **24**, 551-565.e558 (2019).
7. D. N. Perkins, D. J. Pappin, D. M. Creasy, J. S. Cottrell, Probability-based protein identification by searching sequence databases using mass spectrometry data. *Electrophoresis* **20**, 3551-3567 (1999).
8. T. Taus *et al.*, Universal and confident phosphorylation site localization using phosphoRS. *J Proteome Res* **10**, 5354-5362 (2011).
9. K. Clement *et al.*, CRISPResso2 provides accurate and rapid genome editing sequence analysis. *Nat Biotechnol* **37**, 224-226 (2019).
10. P. J. Cock, C. J. Fields, N. Goto, M. L. Heuer, P. M. Rice, The Sanger FASTQ file format for sequences with quality scores, and the Solexa/Illumina FASTQ variants. *Nucleic Acids Res* **38**, 1767-1771 (2010).
11. A. M. Bolger, M. Lohse, B. Usadel, Trimmomatic: a flexible trimmer for Illumina sequence data. *Bioinformatics* **30**, 2114-2120 (2014).
12. H. Li, R. Durbin, Fast and accurate short read alignment with Burrows-Wheeler transform. *Bioinformatics* **25**, 1754-1760 (2009).
13. G. M. Erik Garrison (2012) Haplotype-based variant detection from short-read sequencing. (arXiv), p 3907.
14. P. Cingolani *et al.*, A program for annotating and predicting the effects of single nucleotide polymorphisms, SnpEff: SNPs in the genome of Drosophila melanogaster strain w1118; iso-2; iso-3. *Fly (Austin)* **6**, 80-92 (2012).
15. X. Chen *et al.*, Manta: rapid detection of structural variants and indels for germline and cancer sequencing applications. *Bioinformatics* **32**, 1220-1222 (2016).
16. E. Talevich, A. H. Shain, T. Botton, B. C. Bastian, CNVkit: Genome-Wide Copy Number Detection and Visualization from Targeted DNA Sequencing. *PLoS Comput Biol* **12**, e1004873 (2016).
17. S. Ferrari *et al.*, Efficient gene editing of human long-term hematopoietic stem cells validated by clonal tracking. *Nature Biotechnology* **38**, 1298-1308 (2020).
18. S. Ferrari *et al.*, BAR-Seq clonal tracking of gene-edited cells. *Nat Protoc* **16**, 2991-3025 (2021).
